# Mössbauer Spectroscopy on Antimony Borosulfates Reveals Weak Coordination Behavior

**DOI:** 10.1002/anie.202521198

**Published:** 2025-12-12

**Authors:** Erich Turgunbajew, Gwendolyn Buchner, Aylin Koldemir, Theresa Block, Rainer Pöttgen, David Hemker, Richard Dronskowski, Henning A. Höppe

**Affiliations:** ^1^ Lehrstuhl für Festkörperchemie Universität Augsburg Universitätsstraße 1 86159 Augsburg Germany; ^2^ Institut für Anorganische und Analytische Chemie Universität Münster Corrensstraße 30 48149 Münster Germany; ^3^ Institut für Anorganische Chemie RWTH Aachen University Landoltweg 1 52056 Aachen Germany

**Keywords:** Antimony, Borosulfates, Mössbauer spectroscopy, Thallium, Weak coordination

## Abstract

With M^III^M^I^[B(SO_4_)_2_]_4_ (M^III^ = Bi^3+^, Sb^3+^, Lu^3+^; M^I^ = H_3_O^+^, NO_2_^+^, Li^+^, Na^+^, K^+^, Rb^+^, Cs^+^), we recently described the first modular system within borosulfate chemistry comprising a three‐dimensional anion. Herein, we shed light on the respective series of antimony compounds SbX[B(SO_4_)_2_]_4_ (X = Li^+^, Na^+^, K^+^, Rb^+^, Cs^+^, Ag^+^, Tl^+^, NO^+^, NH_4_^+^). While maintaining the same anionic topology, the compounds crystallize in the space groups *I* $\bar{4}$ (no. 82), *P* $\bar{4}$ (no. 81), and *C*2 (no. 5) and are strongly influenced by the lone pair of antimony as well as the size of the monovalent cations. In the course of this investigation SbX[B_4_O_2_(SO_4_)_6_] (X = Li^+^, Na^+^) were discovered. This borosulfate with a one‐dimensional anion comprising B─O─B bridges crystallizes in the space group *Pnma* (no. 62) and features a new structure type. ^121^Sb Mössbauer spectra revealed negative isomer shifts of almost −22 mm·s^−1^ not observed before and hinting towards a very weak coordination behavior of the borosulfate anion. The spectra are confirmed by DFT calculations. Furthermore, single crystal X‐ray diffraction, infrared spectroscopy, thermal analysis, and temperature programmed X‐ray diffraction experiments were carried out.

## Graphical abstract

The weakly coordinated trivalent antimony atoms show record‐breaking low chemical Mössbauer shifts, and in combination with several monovalent cations including silver, nitrosonium, thallium cations a rich crystal chemistry of closely related but different crystal structures. Sb(NH_4_)[B(SO_4_)_2_]_4_ showed a non‐linear SHG response comparable to that of KDP. Finally, the thermal decomposition beyond 180 °C is discussed.

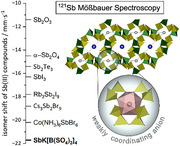

## Introduction

In recent years the interest in borosulfates, a comparably new compound class classified as silicate‐analogous materials, grew substantially.^[^ ^1^ ^]^ Accordingly, borosulfates are related to nitridosilicates, borophosphates, fluorooxoborates, or alumosilicates which all have basic tetrahedral building units *TX*_4_ (*T* = Si, Al, B; *X* = N, O, F) in common.^[^ ^2^, ^3^, ^4^, ^5^ ^]^ Borosulfate anions consist of alternately corner‐sharing borate and sulfate tetrahedra which condensate to form zero‐dimensional groups like in Li_5_[B(SO_4_)_4_],^[^ ^6^ ^]^ chains in K[B(SO_4_)_2_],^[^ ^6^ ^]^ planes in Cd[B_2_(SO_4_)_4_],^[^ ^7^ ^]^ or three‐dimensional networks in Li[B(SO_4_)_2_],^[^ ^8^ ^]^ analogously to silicates. In addition to the conventional B─O─S bridges mentioned above, further connectivity patterns, B─O─B and S─O─S bridges like in Ba[B_2_O(SO_4_)_3_]^[^ ^9^ ^]^ and Ag[B(S_2_O_7_)_2_]^[^ ^10^ ^]^ are formed and further increase the already impressive structural diversity, these violate Loewenstein´s^[^ ^11^ ^]^ and Pauling´s fourth rule,^[^ ^12^ ^]^ respectively, corroborating the limited predictive power of the latter—nicely demonstrated recently.^[^ ^13^ ^]^

Although being comparably recent, with its first representative K_5_[B(SO_4_)_4_] only discovered in 2012, already over a hundred compounds were discovered so far; these not only show a great structural variability but also interesting properties leading to their potential fields of application in phosphors, solid acid electrolytes, or materials with non‐linear optical response (SHG materials).^[^ ^14^, ^15^, ^16^, ^17^, ^18^ ^]^ One striking property of borosulfates is their weak coordination behavior which was observed in multiple studies. For instance, optical spectroscopy was performed on α‐Co_4_[B_2_O(SO_4_)_6_] and Ni_4_[B_2_O(SO_4_)_6_] and revealed a weak ligand field splitting compared to their respective sulfates and chlorides.^[^ ^19^ ^]^ The fluorescence spectra of Ce_2_[B_2_(SO_4_)_6_] showed emission wavelengths in the UV regime, also a strong indication for an extremely weak nephelauxetic effect comparable to that in fluorides.^[^ ^20^ ^]^ In case of Eu_2_[B_2_(SO_4_)_6_], the Van‐Vleck paramagnetism yields a coupling parameter substantially weaker than that of EuF_3_.^[^ ^20^ ^]^ Furthermore, the homopolycation I_4_^2+^ was stabilized in I_4_[B(S_2_O_7_)_2_], recently,^[^ ^21^ ^]^ previously only observed in fluorides.

Recoil‐free nuclear resonance absorption spectroscopy, also known as Mössbauer spectroscopy is a powerful tool to give insights into the oxidation numbers, spin states, and magnetism of solid materials. Furthermore, due to the methods´ strong dependency on the *s*‐electron density one can extract information on the chemical bonding and environment of the considered nuclei meaning its site symmetry, the covalency of a bond, and electronegativity of the ligands. Literature reveals that more than two thirds of the publication on Mössbauer spectroscopy are related to iron and tin nuclei whereas only a very small amount is dedicated to antimony. Sb(V) nuclei typically show isomer shifts ranging from 3 mm·s^−1^ in NaSbF_6_ to −4 mm·s^−1^ in SbCl_2_F_3_ gradually shifting from ionic to more covalent bonding.^[^ ^22^ ^]^ The isomer shift in Sb(III) nuclei is normally found at lower values between −10 and −16 mm·s^−1^ where a lower value is associated with a weaker, more ionic coordination. In some rare examples isomer shifts below −16 mm·s^−1^ are observed, with Co(NH_3_)_6_SbCl_6_ reaching a value of −20.2 mm·s^−1^, the lowest value achieved so far to the best of our knowledge.^[^ ^23^ ^]^ As mentioned above, borosulfate anions are known to be weakly coordinating and hence are also expected to produce low isomer shift values. This coordination weakens with increasing condensation degree of the anion and increasing sulfate content.

In this contribution we elucidate the crystal structures of new antimony borosulfates, also containing thallium and nitrosonium cations, and not known in borosulfate chemistry so far. We thoroughly discuss the structural influence of the monovalent cations on the crystal structures and infrared spectra. By employing ^121^Sb Mössbauer spectroscopy, we shed light not only on the oxidation state of antimony, but the coordination strength of borosulfates in general, supported by density functional theory (DFT) calculations. The characterization of this series is concluded by thermogravimetric analysis (TGA), infrared (IR) spectroscopy, and temperature programmed powder X‐ray diffraction (TPPXRD).

## Results and Discussion

### Crystal Structures of SbX[B(SO_4_)_2_]_4_ (X = Li^+^, Na^+^, K^+^, Rb^+^, Cs^+^, Ag^+^, Tl^+^, NO^+^, NH_4_^+^)

The series of antimony borosulfates SbX[B(SO_4_)_2_]_4_ (X = Li^+^, Na^+^, K^+^, Rb^+^, Cs^+^, Ag^+^, Tl^+^, NO^+^, NH_4_^+^) crystallizes in the space groups *I* $\bar{4}$ (no. 82), *P* $\bar{4}$ (no. 81) and *C*2 (no. 5) as listed in Table 1. Details can be found in the supplementary information (Tables S1, S2).^[^ ^24^ ^]^ The evolution of space groups with altering monovalent cation does not follow a strict pattern as was previously observed in the structurally related bismuth and lutetium series M^III^M^I^[B(SO_4_)_2_]_4_ (M^III^ = Bi^3+^, Lu^3+^; M^I^  =  Li^+^, Na^+^, K^+^, Rb^+^, Cs^+^) where the choice of the space group was mainly influenced by the size of the monovalent cations. After reaching a threshold, along with a small distortion of the network a symmetry descent was induced and the structures consequently crystallized in space group *P* $\bar{4}$ instead of *I* $\bar{4}$.^[^ ^25^ ^]^ Therefore, additional impact factors must be responsible for the observed space group development along the series. The interplay between electronic and sterical effects like the expression of a lone pair and the sizes of both the mono‐ and trivalent cation apparently play a crucial role.

**Table 1:** Space groups of SbX[B(SO_4_)_2_]_4_ (X = Li^+^, Na^+^, K^+^, Rb^+^, Cs^+^, Ag^+^, Tl^+^, NO^+^, NH_4_^+^) in correlation with the ionic radii of the monovalent cations.

Compound	r^[^ ^8^ ^]^(X)/pm	Space group
SbLi[B(SO_4_)_2_]_4_	92	*I* $\bar{4}$
SbNa[B(SO_4_)_2_]_4_	118	*I* $\bar{4}$
SbK[B(SO_4_)_2_]_4_	151	*C*2
SbRb[B(SO_4_)_2_]_4_	161	*C*2
SbCs[B(SO_4_)_2_]_4_	174	*P* $\bar{4}$
SbAg[B(SO_4_)_2_]_4_	128	*P* $\bar{4}$
SbTl[B(SO_4_)_2_]_4_	159	*C*2
Sb(NO)[B(SO_4_)_2_]_4_	–	*C*2
Sb(NH_4_)[[B(SO_4_)_2_]_4_	154	*C*2

**Figure 1:**
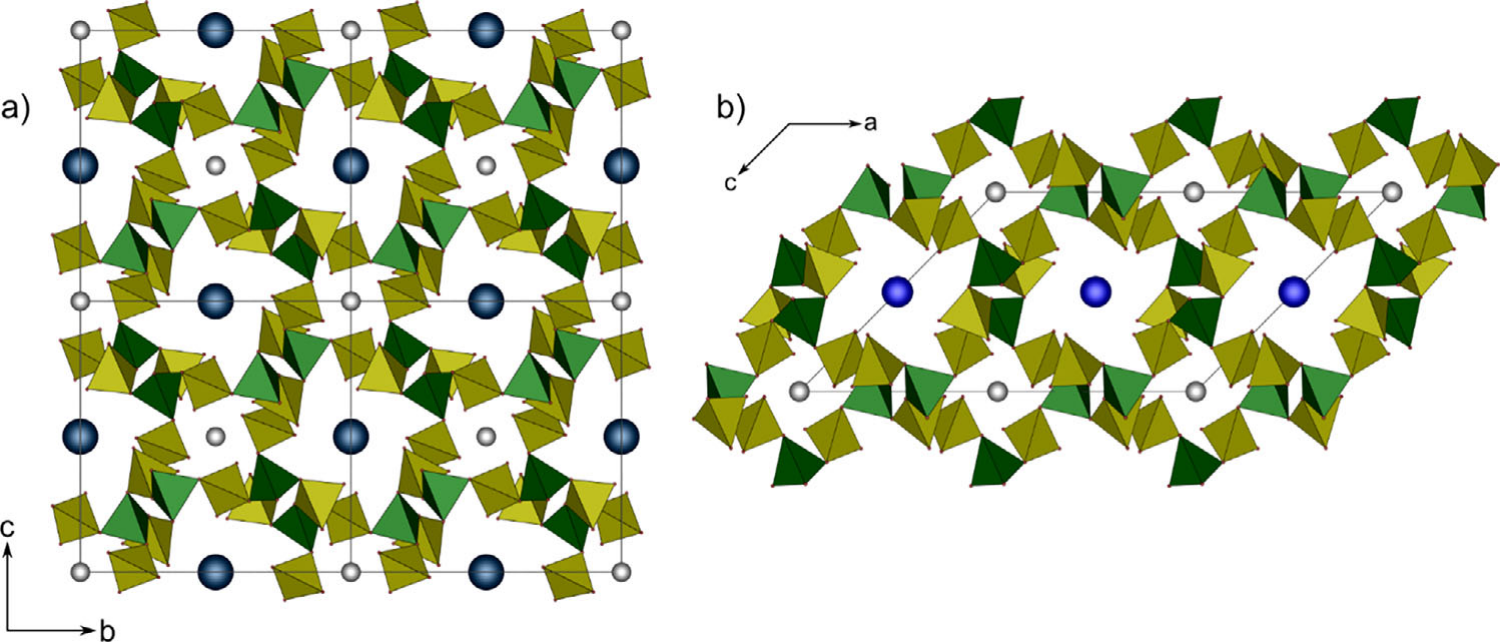
Unit cells of a) SbCs[B(SO_4_)_2_]_4_ and b) SbK[B(SO_4_)_2_]_4_ representing the tetragonally and monoclinically crystallizing compounds; antimony shown as gray spheres, cesium in dark blue, potassium in blue; sulfate tetrahedra are shown in yellow, borate in green.

**Figure 2:**
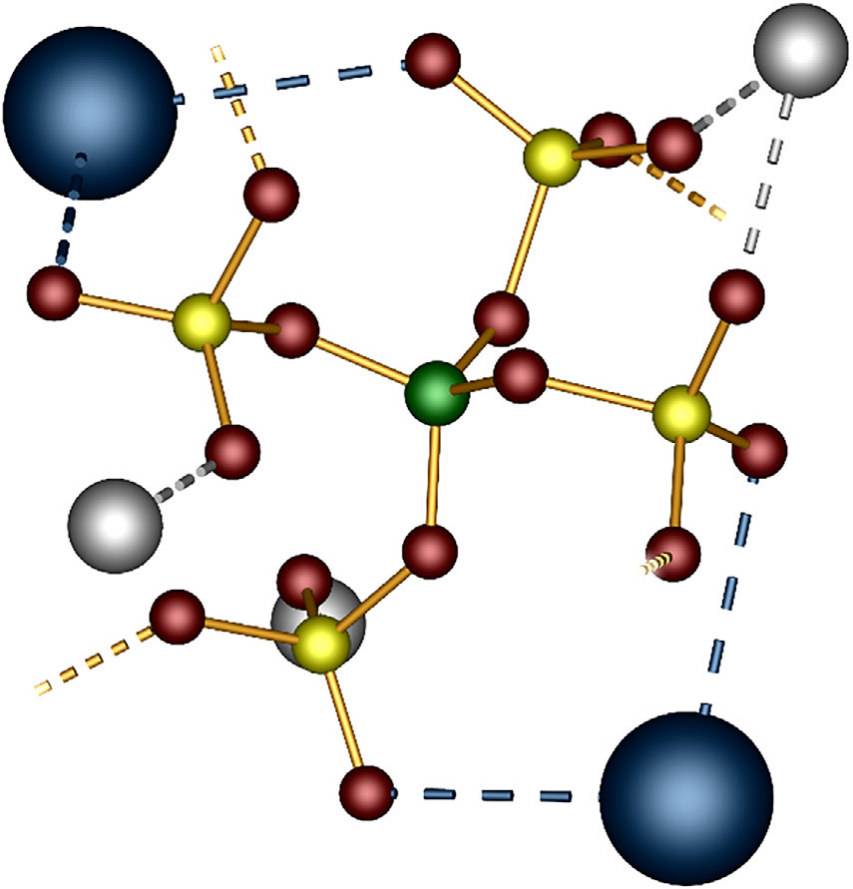
B(SO_4_)_4_ moiety in SbX[B(SO_4_)_2_]_4_ (X = Li^+^, Na^+^, K^+^, Rb^+^, Cs^+^, Ag^+^, Tl^+^, NO^+^, NH_4_^+^); dotted yellow lines indicate the connection to further boron atoms; boron in green, sulfur in yellow, oxygen in red, cesium in blue, antimony in gray.

Although the title compounds crystallize in different space groups, their anion topology is the same and can therefore be described collectively. The anion is built up by vertex connected borate and sulfate tetrahedra leading to a three‐dimensional network, where all boron atoms are surrounded more or less tetrahedrally by sulfate ions which themselves bridge two boron atoms each. Accordingly, a tectosilicate analogous anion [B(SO_4_)_2_]^−^ is achieved. Channels extend along the c‐ and b‐direction, respectively (Figure 1). Figure 2 shows a section of the structure displaying a B(SO_4_)_4_ moiety and its connection toward mono‐ and trivalent cations. The B–O distances range between 1.45–1.47 Å, the bridging S–O_br_ distances between 1.51–1.54 Å, and with the terminal S─O_term_ bonds toward coordinating the monovalent cations lie between 1.40–1.42 Å, those coordinating to the antimony atoms between 1.45 and 1.46 Å, respectively. The distances are in accordance with the sum of the ionic radii and other borosulfates.^[^ ^19^, ^25^, ^26^, ^27^ ^]^ Further details concerning the bond lengths are summarized in Table S6. With deviations smaller than one percent, all tetrahedra can be classified as regular (Table S9). Additional calculations based on the MAPLE concept^[^ ^28^, ^29^, ^30^, ^31^, ^32^ ^]^ were performed and confirm the electrostatic consistency of all compounds (Table S4).

**Figure 3:**
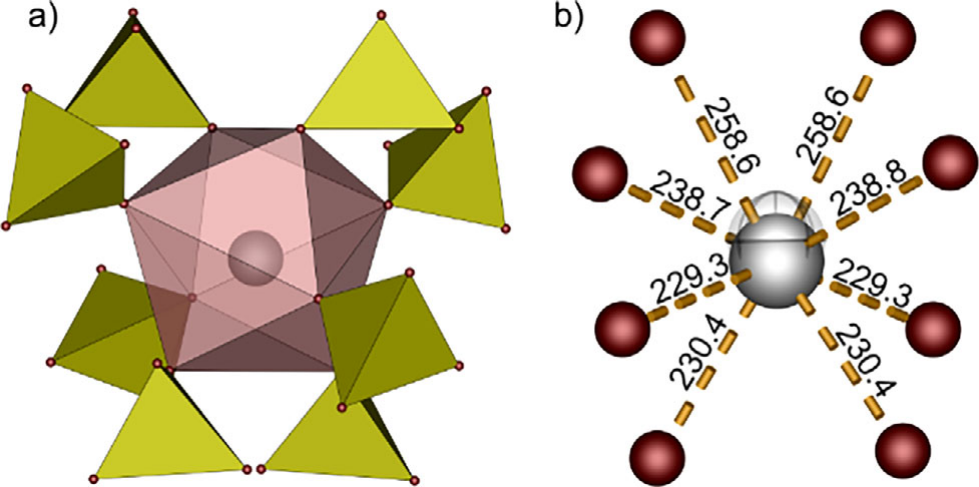
Coordination environment of antimony in SbK[B(SO_4_)_2_]_4_ viewed along [001]: a) square antiprismatic SbO_8_ coordination polyhedron formed by eight sulfate groups (yellow). b) same SbO_8_ polyhedron with bond distances (in pm) and the centroid (semitransparent octant) revealing the direction of the expressed lone pair. antimony in gray; oxygen in red.

Charge compensation is realized by mono‐ and trivalent cations residing in the channels. The latter are located within the narrower channels and are coordinated distorted square antiprismatically by eight terminal oxygen atoms stemming exclusively from sulfate groups (Figure 3a). Like bismuth, antimony in its trivalent state is also considered an *s* ^2^‐cation and can therefore express a lone pair effect which typically is more pronounced in antimony compounds compared to their related less electronegative bismuth homologues.^[^ ^33^, ^34^ ^]^ To quantify the lone pair influence we used a geometrical approach suggested by Hämmer et al.,^[^ ^35^ ^]^ which is based on calculations developed by Balić‐Žunić and Mackovicky creating an enclosing sphere based on all ligands.^[^ ^36^, ^37^ ^]^ Accordingly, the deviation of its centroid quantifies the lone pair expression and reveals its direction (Figure 3b). The results are presented in Table S5. The lithium, sodium, and cesium compounds show almost no deviation and crystallize in the tetragonal space groups *I* $\bar{4}$ and *P* $\bar{4}$. For the remaining compounds, deviations between 12 and 19 pm are found and go along with space group *C*2. However, the respective silver compound is an exception to the rule and crystallizes in space group *P* $\bar{4}$ despite a centroid deviation of 25 pm. Besides a comparably strong expressed lone pair, further aspects seem to influence the structures and should be considered.

**Figure 4:**
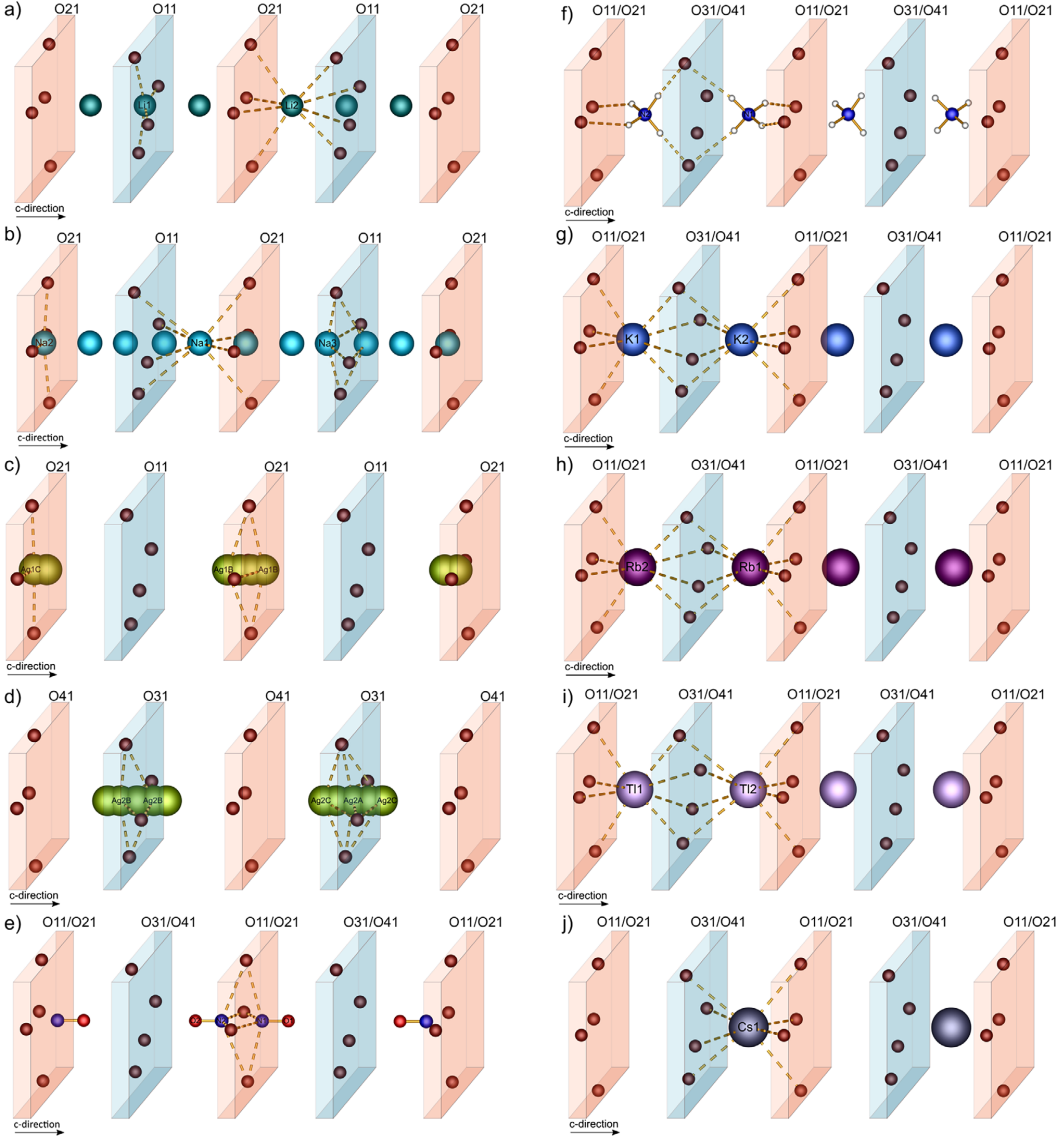
Position and coordination of a) lithium, b) sodium, c) silver (channel 1) and d) silver (channel 2), e) nitrosonium, f) ammonium, g) potassium, h) rubidium, i) thallium, j) cesium ions with planes belonging to different oxygen species marked in blue and red. The silver cations within the respective compound were refined isotropically with 12 positions. For a better overview, positions with a smaller fractional occupation factor than 5% were omitted.

The monovalent cations are situated in the wider channels. Their position can effectively be described by defining two planes, spanned by terminal oxygen atoms stemming from sulfate tetrahedra pointing into the channel (Figure 4). Depending on the cation´s ionic radius several positions within the channels are plausible, either within a plane, in between, or both simultaneously which can be realized by disorder. In contrast to the related bismuth and lutetium compounds which were predominantly dominated by the size of the mono‐ and trivalent cation, here, the combination of a smaller ionic radius and expected stronger lone pair effect of antimony, together with the size and electronic configuration of the respective monovalent cation are responsible for the observed structural changes.^[^ ^25^ ^]^ Hence, both effects need to be treated simultaneously. Determined by single‐crystal XRD data, lithium shows a disorder and was refined with an occupation of 60% toward the Li2 position, hence, preferably a position between the layers. The same trend was observed for sodium, occupying three positions with the highest probability of 50% to find sodium on a Na1 position, also between layers. Both structures crystallize in the tetragonal space group *I* $\bar{4}$ and show a quenched lone pair of the antimony cation. Contrarily to the remaining compounds of this series, SbX[B(SO_4_)_2_]_4_ (X = Li^+^, Na^+^) were not synthesized phase purely but were obtained as a side phase of the newly discovered antimony borosulfates SbX[B_4_O_2_(SO_4_)_6_] (X = Li^+^, Na^+^). Powder data of all compounds are presented in the supplements in Figures S3, S4. Although all reactions were driven under the same reaction conditions, the impact of the lithium and sodium ions seem to be not strong enough to stabilize the three‐dimensional phase. As a result, the compounds crystallize in the thermodynamically more stable phase SbX[B_4_O_2_(SO_4_)_6_] (X = Li^+^, Na^+^) which will be discussed below.

The silver atoms of the respective silver compound were refined isotropically due to an even stronger disorder with 12 individual positions in total. The highest occupied position (Ag1c) is located within the layer with a fractional occupation factor of 33%. Even though sodium shares a similar ionic radius (r^[4]^ (Na) = 99 pm and r^[4]^(Ag) = 100 pm) strikingly both ions prefer different positions within the channels and crystallize in different space groups. This trend was also observed for the respective bismuth compounds; but here no change in symmetry was reported.^[^ ^38^ ^]^ The occupation of silver atoms (Ag1c) within the layers causes the plane to flatten to an almost square planar coordination environment. Consequently, two distinguishable channels are formed (Figure 4). Although silver ions are known to behave frequently like alkali cations, due to their electronic configuration they also often show a different behavior and typically prefer lower coordination numbers.^[^ ^39^ ^]^ Interestingly, this compound is the only one of all structures belonging to this structure type which has to be described by the second possible set‐up of *P* $\bar{4}$ in which we end up with a unique antimony position instead of commonly two. Except for the cesium compound, which also crystallizes in space group *P* $\bar{4}$ with two unique antimony positions, the remaining compounds in this series all crystallize in the monoclinic space group *C*2. It seems that we enter a region where the size of the monovalent cation enhances the expression of the lone pair and a distortion of the framework to loosen the four‐fold symmetry. A further trend can be observed whereby an increasing ionic radius comes along with a more expressed lone pair (Table S5). To confirm the non‐centrosymmetric space group SHG measurements were performed on Sb(NH_4_)[B(SO_4_)_2_]_4_. The non‐linear response of Sb(NH_4_)[B(SO_4_)_2_]_4_ was comparable to potassium dihydrogenphosphate (KDP) and hence confirms the non‐centrosymmetric space group (Figure S12).

In this series of antimony borosulfates an unambiguous determination of the correct space groups often was challenging as the structural refinements offered several space groups with similar *R* values. A structural validation in PLATON^[^ ^40^, ^41^, ^42^ ^]^ consistently suggested a refinement in the highest possible space group *I* $\bar{4}$. A direct comparison between the experimental and simulated diffraction patterns turned out to be a helpful tool to identify the proper space group. Two striking features can be observed which occur due to the symmetry descent to the lower symmetric space groups *P* $\bar{4}$ and *C*2. Throughout the series, a symmetry lowering to the monoclinic space group *C*2 leads to an obvious splitting of reflections. This splitting is illustrated for SbK[B(SO_4_)_2_]_4_ where, e.g., the reflections around 23.8° and 25.1° are split into four and two reflections, respectively, compared to the higher symmetric refinement in space group *I* $\bar{4}$ (Figure S5). Similarly, by comparing the crystal structure refinements of SbAg[B(SO_4_)_2_]_4_ in space groups *P* $\bar{4}$ and *I* $\bar{4}$, consistently additional reflections around 11° and 19° 2*θ* appear and hence, unambiguously specifies the proper space group (Figure S6).

### Crystal Structure of SbNa[B_4_O_2_(SO_4_)_6_]

**Figure 5:**
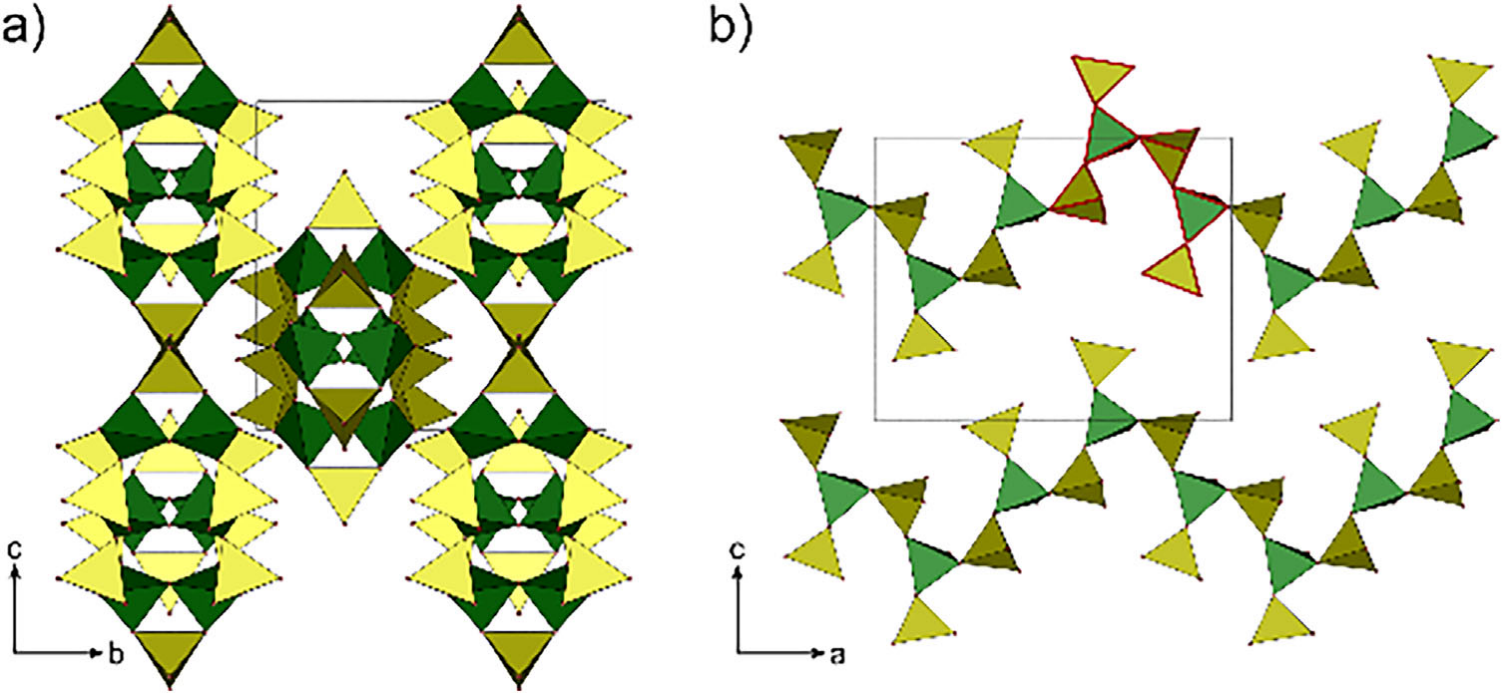
Unit cell of SbNa[B_4_O_2_(SO_4_)_6_] viewed along the crystallographic a) a‐direction and b) b‐direction. For better clarity of the anionic structure the cations are neglected. The FBU [B_4_O_2_(SO_4_)_6_]^4−^ is highlighted with red lines; sulfate tetrahedra in yellow; borate tetrahedra in green.

**Figure 6:**
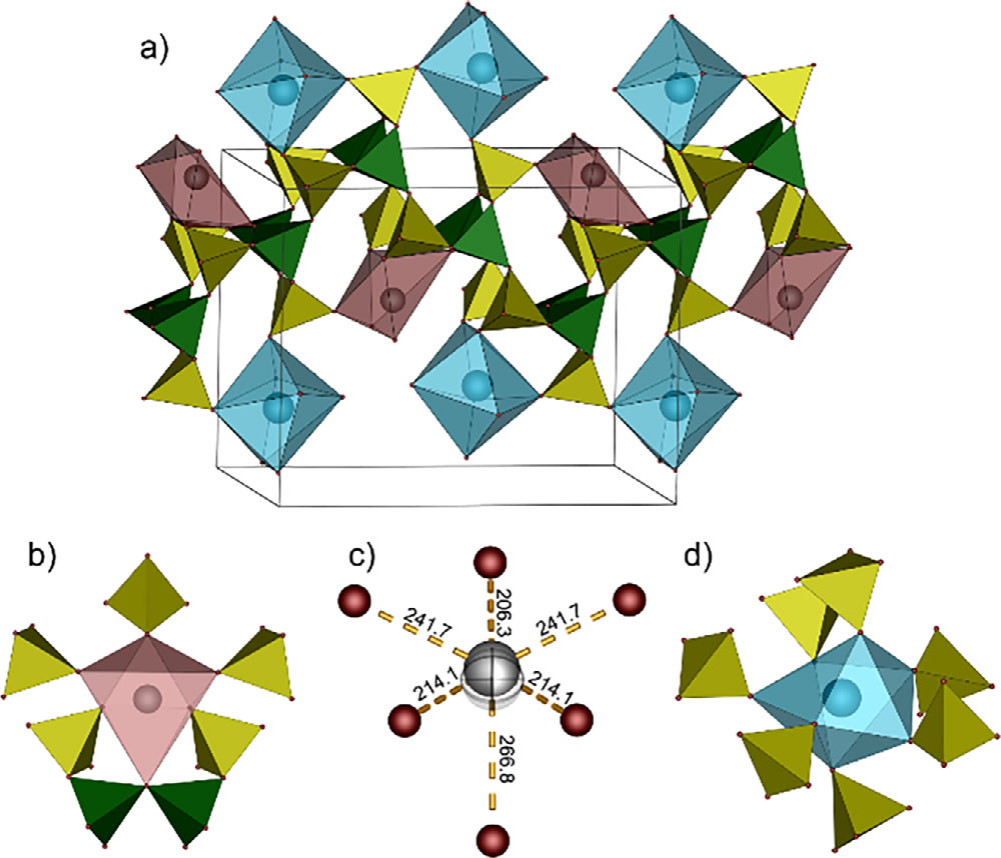
Section of the anionic chain in SbNa[B_4_O_2_(SO_4_)_6_] with the coordination environment of both cations: a) zig–zag chains with antimony cations (red polyhedra) positioned within and sodium cations (blue polyhedra) above and below the chains. b) Coordination environment of antimony cations (red polyhedron). c) Graphical illustration of the antimony lone pair activity in the SbO_6_ polyhedron including the centroid (semitransparent sphere) and the respective bond lengths in pm; antimony in gray, oxygen in red. d) Coordination environment of sodium cations (blue polyhedron).

SbNa[B_4_O_2_(SO_4_)_6_] crystallizes in a new structure type in the orthorhombic space group *Pnma* (no. 62) with four formula units per unit cell (Figure 5). Further details may be found in the supplementary information (Table S3).^[^ ^24^ ^]^ The structure comprises two parallel running chains of alternating borate and sulfate tetrahedra running along the crystallographic a‐direction. Both zig–zag shaped chains are connected via two common borate corners as well as one bridging sulfate tetrahedron forming *dreier* single rings with the latter (Figure S1). Therefore, according to Liebau´s nomenclature, the topology can be described as loop‐branched *achter* double chains.^[^ ^43^ ^]^ Even though, the ribbon like chains with B─O─B bridges—hence, classified as unconventional borosulfate—show similarities to the compounds X[B_2_O(SO_4_)_3_] (X = Sr^2+^, Pb^2+^, Ba^2+^, Cd^2+^)^[^ ^9^, ^44^, ^45^ ^]^ featuring a [B_4_O_2_(SO_4_)_6_]^4−^ unit, this structure here offers a new fundamental building unit (FBU) not known for borosulfates, so far (Figures 5b and S1). This FBU can be viewed as a modification of the FBU found in Cd[B_2_O(SO_4_)_3_] where along the chains sulfate tetrahedra that are part of the *dreier* ring alternately point up and downwards. In SbNa[B_4_O_2_(SO_4_)_6_], this scheme is modified to two sulfate units pointing up followed by two pointing down (Figure S2). Followed by this description, the loop‐branched *vierer* double chains in Cd[B_2_O(SO_4_)_3_] are extended to loop‐branched *achter* double chains in SbNa[B_4_O_2_(SO_4_)_6_] (See Figure 6).

The respective bond lengths in the borate tetrahedra B–O_br_ range between 139–142 pm in B─O─B bridges and extend to 147–153 pm for B─O─S bridges due to electrostatic reasons. The sulfate tetrahedra reveal S─O bond distances ranging from 150–154 pm in bridging S─O_br_ bonds. Furthermore, the bond distances of terminal oxygen atoms S─O_term_ coordinating antimony ions ranging from 143–148 pm are slightly longer compared to the ones coordinating the sodium cation with bond lengths of 141–143 pm, respectively. These results agree well with the distances observed earlier in the described series of three‐dimensional antimony borosulfates comprising mono‐ and trivalent cations simultaneously and unconventional borosulfates forming B─O─B bridges.^[^ ^9^, ^25^, ^44^, ^45^ ^]^

Sodium occupies a position between the chains and is coordinated by seven terminal oxygen atoms stemming from monodentate sulfate tetrahedra forming a distorted pentagonal bipyramid (Figure 5d). The bond distances agree well with sum of ionic radii (Table S7).

Antimony is situated within the ribbons coordinated irregularly six‐fold by five terminal oxygen atoms of monodentate sulfate units and a bridging oxygen atom of the B─O─B bridge units (Figure 5b). Calculations based on the MAPLE concept confirmed the coordination number (Table S8).

### ^121^Sb Mössbauer Spectroscopy

**Figure 7:**
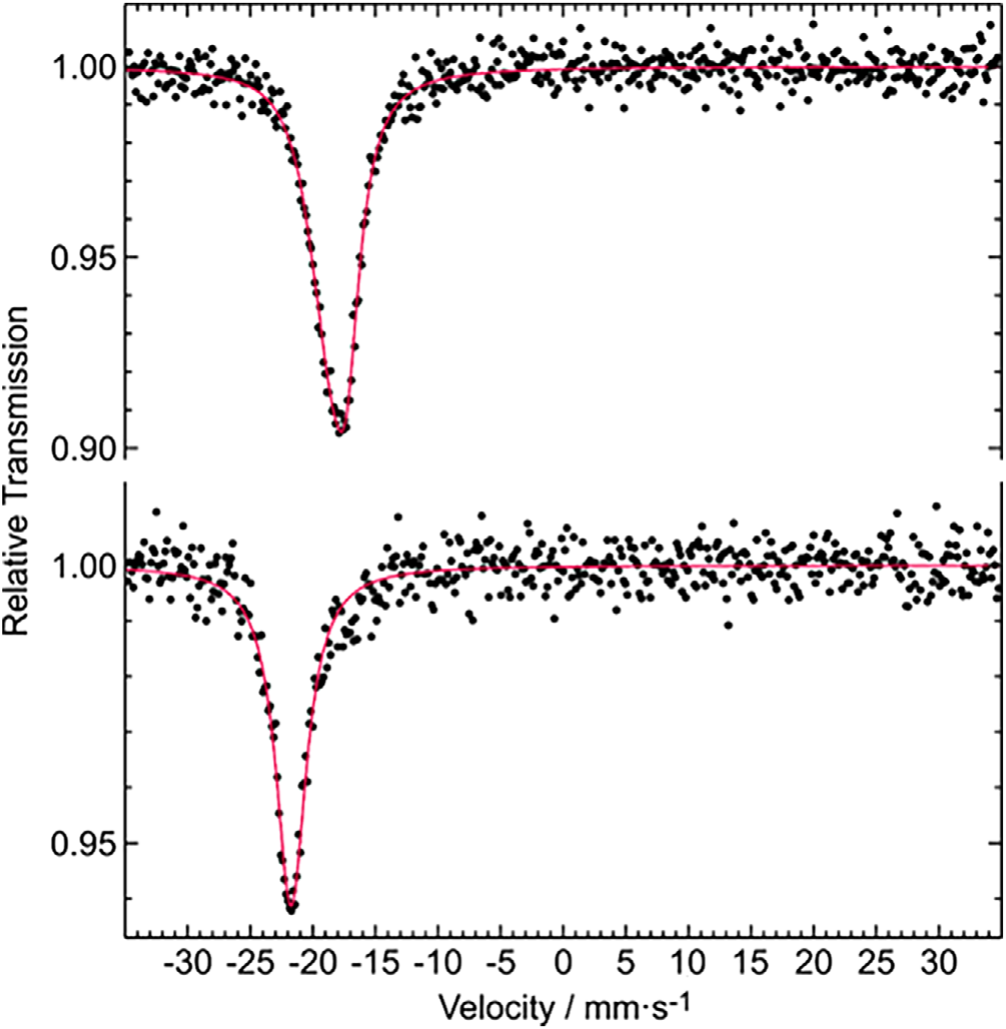
Experimental (data points) and simulated (red line) ^121^Sb Mössbauer spectra of SbNa[B_4_O_2_(SO_4_)_6_] (top) and Sb(NO)[B(SO_4_)_2_]_4_ (bottom) at 78 K.

Isomer shifts determined from Mössbauer spectra mirror the electron density at the studied nuclei. We have therefore collected ^121^Sb spectra of the antimony‐based borosulfates in order to get more experimental information on the bonding situation. The fitting parameters for the different antimony‐based borosulfates are listed in Table 2. The 78 K spectra of Sb(NH_4_)[B(SO_4_)_2_]_4_ and SbNa[B_4_O_2_(SO_4_)_6_] are presented as examples in Figure 7. A compilation of the remaining spectra is given in Figure S13 in the supplementary information. Within the combined standard uncertainties of the fitting parameters, all borosulfates show similar isomer shifts. This is underpinned with a grayish line in Figure S13. There is no noticeable dependence with respect to the monovalent cation. The borosulfates SbX[B(SO_4_)_2_]_4_ (X = NO^+^, NH_4_^+^, K^+^, Rb^+^, Cs^+^) show single signals in agreement with the unique crystallographic antimony sites (2*a* in space group *C*2, site symmetry 2). The striking features of these spectra are the extremely negative isomer shifts around −22 mm·s^−1^ which cannot be compared to any other antimony compound. The ionic formula splitting for Sb(NH_4_)[B(SO_4_)_2_]_4_, i.e., (NH_4_^+I^Sb^+III^B_4_^+III^S_8_^+VI^N^−III^O_32_^−II^) clearly points to trivalent antinomy; however, the usual isomer shift range for trivalent antimony compounds in literature is from about −10 to more or less −16 mm·s^−1^,^[^ ^22^, ^46^, ^47^, ^48^ ^]^ to around −18 mm·s^−1^ for very few exceptions (vide infra). The very low isomer shift of the antimony atoms in these borosulfates indicates a high *s*‐electron density, due to the weak coordination behavior of the borosulfate anions (Sb@O_8_ square antiprism formed by four different borosulfate anions).^[^ ^1^ ^]^ Although the antimony atoms have only site symmetry 2 and different Sb─O bond lengths, the spectra could be well reproduced without electric quadrupole splitting. A reason for this could be that there is no significant distortion of the *s*‐electron density at the antimony nuclei because of the weak coordinating borosulfate anions. The experimental line widths are in the usual range. In order to rule out any irregularity from the instrument, also an *α*‐Sb^III^Sb^V^O_4_ sample was measured. The fitting parameters (Table 2) are in good agreement with literature values.^[^ ^49^, ^50^ ^]^

**Table 2:** Fitting parameters of ^121^Sb Mössbauer spectroscopic measurements at 78 K. *δ* = isomer shift, Δ*E*_Q_ ^a)^ = electric quadrupole splitting, *Γ* = experimental line width. Parameters marked with an asterisk were kept fixed during the fitting procedure.

compound		*δ* (mm·s^−1^)	Δ*E*_Q_ (mm·s^−1^)	*Γ* (mm·s^−1^)
*α*‐Sb_2_O_4_	Sb(V)	0.36(2)	−2.6(4)	3.2(1)
	Sb(III)	−14.62(4)	8.3(2)	3.2(1)
SbNa[B_4_O_2_(SO_4_)_6_]		−18.17(3)	4.8(2)	2.8(1)
Sb(NO)[B(SO_4_)_2_]_4_		−21.74(2)	0*	3.00(8)
Sb(NH_4_)[B(SO_4_)_2_]_4_		−21.73(1)	0*	3.0(1)
SbK[B(SO_4_)_2_]_4_		−21.60(3)	0*	2.63(9)
SbRb[B(SO_4_)_2_]_4_		−21.44(4)	0*	2.9(1)
SbCs[B(SO_4_)_2_]_4_		−21.76(4)	0*	2.8(2)

^a)^ Δ*E*_Q_ with *eQV_ZZ_*/2.

In contrast, the borosulfate anion in SbNa[B_4_O_2_(SO_4_)_6_] exhibits a slightly stronger coordination of the antimony cation which is reflected in a less negative isomer shift of −18.2(1) mm·s^−1^. We assume that this difference in coordination strength is predominantly affected by two factors: the smaller coordination number of six in SbNa[B_4_O_2_(SO_4_)_6_] compared to eight in SbX[B(SO_4_)_2_]_4_ (X = NO^+^, NH_4_^+^, K^+^, Rb^+^, Cs^+^) results in shorter average bond distances and hence higher coordination strength (Tables S8, S10). Second, in the three‐dimensional structure antimony is exclusively surrounded by sulfate units while in the inosilicate‐analogue, the cation is additionally coordinated by BO_4_‐units. Furthermore, a weak quadrupole splitting of 4.8(2) mm·s^−1^ was refined. Hence, the electronic situation of antimony in SbNa[B_4_O_2_(SO_4_)_6_] should be comparable to the chlorides Cs_3_Sb_2_Cl_9_ (*δ* = −9.67 mm·s^−1^ vs. InSb, i.e., −18.27 mm·s^−1^ rel. to Ba^121^SnO_3_) and (n‐C_4_H_9_NH_3_)_3_Sb_2_Cl_9_ (*δ* = −9.14 mm·s^−1^ rel. to InSb, i.e., −17.76 mm·s−1 rel. to Ba^121^SnO_3_)^[^ ^51^, ^52^ ^]^ which exhibit larger chloridoantimonate anions.^[^ ^52^ ^]^

### Density Functional Theory (DFT)

**Figure 8:**
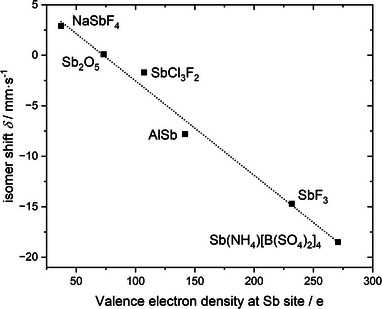
Linear correlation between the experimental Mössbauer ^121^Sb isomer shifts and the calculated valence electron densities.

Figure 8 depicts the expected linear dependency of the isomer shift on the electron density at the nuclear site. Extrapolating the linear trend to the density of Sb(NH_4_)[B(SO_4_)_2_]_4_, which is calculated to be 270.79 e, results in an isomer shift of −18.5 mm·s^−1^ based on a modified Becke–Johnson functional; the standard PBE functional yields a 4% smaller shift. Although the −18.5 mm·s^−1^ differs from the experimental value of ‐21.73 mm·s^−1^, it follows the qualitative trend, showing an exceptionally large negative isomer shift which mainly goes back to the role of the Sb5*s* orbital, the latter contributing −16.0 mm·s^−1^. Differences between experiment and simulation might be a result of density functional theories self‐interaction error, yielding an underestimation of electron densities at high‐density points, such as the nuclear sites. Evident from the linear correlation, this underestimation of the electron density results in higher (less negative) isomer shifts. Attempts to remedy this issue by utilizing hybrid functionals were performed, however, deemed unfeasible after extrapolation of computational runtime.

### Fourier‐Transformed Infrared Spectroscopy

**Figure 9:**
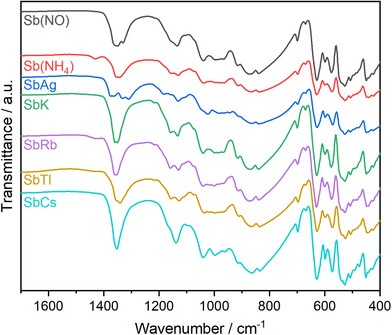
IR spectra of SbX[B(SO_4_)_2_]_4_ (X = K^+^, Rb^+^, Cs^+^, Ag^+^, Tl^+^, NO^+^, NH_4_^+^) in the range between 1700–400 cm^−1^.

Figure 9 displays the infrared spectra of SbX[B(SO_4_)_2_]_4_ (X = K^+^, Rb^+^, Cs^+^, Ag^+^, Tl^+^, NO^+^, NH_4_^+^) (see full spectrum in Figure S11) between 1500 and 400 cm^−1^ which is the typical region for vibrations of borate and sulfate tetrahedra. In a recent contribution on the bismuth homologues BiX[B(SO_4_)_2_]_4_ (X = NH_4_^+^, NO_2_^+^, NO^+^) DFT calculations were conducted and gave access to simulated IR spectra. Consequently, a direct band assignment due to a topologically similar anionic framework of the titled compounds is feasible.^[^ ^53^ ^]^ Upon closer examination of Figure 9, it becomes evident that all spectra are almost identical except for two regions. First, whenever polyatomic monovalent cations are present, additional characteristic bands between 1400 and 4000 cm^−1^ arise like in the case of ammonium and nitrosonium ions. Ammonium shows two typical bands located at 3292 and 1430 cm^−1^ caused by an asymmetric stretching mode ν_as_ (N─H) and an asymmetric deformation mode δ_as_ (N─H), respectively, whereas the nitrosonium cation is characterized by a stretching mode ν (N─O) around 2296 cm^−1^. These results are well in agreement with the performed DFT calculation and further literature.^[^ ^53^, ^54^ ^]^ In addition, each monovalent cation introduced into this system gives rise to a varying impact on the symmetric stretching modes ν_s_ (S─O) of terminal sulfur oxygen bonds around 1350 cm^−1^. A possible explanation for this observed phenomenon was analyzed in the earlier mentioned contribution and related the observed differences to the different positions of the monovalent cations within the channel. Figure 4 displays all monovalent cations located within a one‐dimensional channel extending along b‐direction (c‐direction in tetragonal space groups) which is built up by terminal oxygen atoms. Accordingly, two planes can be defined. Generally, three cases can be distinguished where a cation can either be located within, between two planes or occupy both positions simultaneously which can be achieved by disorder. It was shown that whenever a cation is located between the planes like it is the case of BiCs[B(SO_4_)_2_]_4_ only a single band is observed whereas two bands are present whenever a cation is located within a plane as present in Bi(NO_2_)[B(SO_4_)_2_]_4_.^[^ ^25^ ^]^ Therein, bands of coordinated oxygen shift toward lower and accordingly non‐coordinated toward higher wavenumbers. An additional splitting may be attributed to further occupied positions in the channel which is caused by disorder or by a symmetry related splitting of degenerate vibrations. For a more thorough discussion on this topic we refer to the contribution on the analogous bismuth metal borosulfates BiX[B(SO_4_)_2_]_4_ (X = NH_4_^+^, NO^+^, NO_2_^+^, H_3_O^+^).^[^ ^53^ ^]^ The remaining bands are identical and can be treated analogously. Asymmetric stretching vibrations ν_as_ (B─O) located at 1180 and 1150 cm^−1^ are followed by symmetric stretching vibrations ν_s_ (B─O) occurring between 1040–990 cm^−1^. Symmetric stretching vibrations ν_s_ (S─O) can be found between 930 and 920 cm^−1^. Finally, the region between 700 to 400 cm^−1^ can be assigned to bending vibrations δ (O─S─O, O─B─O, S─O─B).

### Thermogravimetric Analysis

Thermal decomposition processes in borosulfate chemistry often yield further borosulfates; these are sometimes even exclusively accessible through the latter.^[^ ^10^, ^45^ ^]^ In the phase diagram BaO─B_2_O_3_─SO_3_ a conversion from S─O─S over B─O─S to B─O─B bridges was observed where Ba[B(S_2_O_7_)_2_]_2_ initially decomposes to Ba[B_2_(SO_4_)_4_] and finally leads to Ba[B_2_O(SO_4_)_3_].^[^ ^55^ ^]^ If no further borosulfates are formed, the decomposition typically results in the corresponding sulfate and later at even higher temperatures in the metal oxide. In some cases, the metal oxide reacts with B_2_O_3_ to form the respective borate.^[^ ^20^ ^]^ Thermal decomposition studies on the analogue bismuth compounds BiX[B(SO_4_)_2_]_4_ (X = Li^+^, Na^+^, K^+^, Rb^+^, Cs^+^, Ag^+^, Tl^+^) were the first thermal decomposition study of borosulfates containing two differently charged cations.^[^ ^38^ ^]^ It was observed that all compounds are stable up to 250 °C and followed the same reaction scheme in which initially their respective sulfates and eventually their oxides are formed. The decomposition unfortunately did not lead to the formation of new borosulfates containing both cations forming B─O─B bridges as it is observed in the case mentioned above. A comparison to the antimony series reveals clear differences. Not only do the antimony compounds decompose at lower temperatures around 180 °C, but also show a more complex and distinctive degradation process, which is specific to almost every combination of mono‐ and trivalent cations.

**Figure 10:**
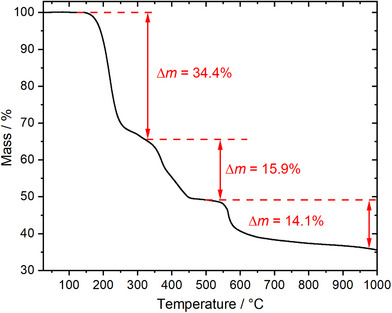
Thermal analysis of SbK[B(SO_4_)_2_]_4_ under nitrogen atmosphere and a heating rate of 5 K·min^−1^.

Among the titled compounds, only the decomposition of SbK[B(SO_4_)_2_]_4_ is well understood and accordingly is characterized by thermogravimetric analysis TGA and temperature programmed powder X‐ray‐diffraction TPPXRD. SbK[B(SO_4_)_2_]_4_ decomposes in a three‐step process (Figure 10). In the first step, accompanied by the release of 4.5 mol SO_3_, Sb_2_(SO_4_)_3_, and the borosulfate K[B(SO_4_)_2_] (Δ*m*_calc_ = 37.1 wt%) are formed which was also confirmed in situ by TPPXRD (Figure S7):

$$\begin{array}{cc} & \text{SbK}{\left[B{\left({\text{SO}}_{4}\right)}_{2}\right]}_{4}\to 0.5{\text{Sb}}_{2}{\left({\text{SO}}_{4}\right)}_{3}+K\left[B{\left({\text{SO}}_{4}\right)}_{2}\right]\\ & \;+1.5B_{2}O_{3}+4.5{\text{SO}}_{3} \end{array}$$

Afterwards the borosulfate K[B(SO_4_)_2_] further decomposes to form K_2_SO_4_ releasing one further SO_3_ molecule (Δ*m*_calc_ = 12.4 wt%):

$$\begin{array}{cc} & 0.5\;{\text{Sb}}_{2}{\left({\text{SO}}_{4}\right)}_{3}+K\left[B{\left({\text{SO}}_{4}\right)}_{2}\right]+1.5\;B_{2}O_{3}\to 0.5\;{\text{Sb}}_{2}{\left({\text{SO}}_{4}\right)}_{3}\\ & \;+0.5\;K_{2}{\text{SO}}_{4}+2\;B_{2}O_{3}+\;{\text{SO}}_{3} \end{array}$$

The observed deviation between the experimental and calculated mass losses for the first two steps (Δ*m*_obs_ = 34.4 wt%, 15.9 wt%) may be explained by a slow conversion rate toward the borosulfate K[B(SO_4_)_2_] and Sb_2_(SO_4_)_3_ which at 300 °C is not fully completed yet and, consequently, influences the mass loss for the second step. An overall observed mass loss in the first two steps of 50.4 wt% agrees well with the calculated 49.4 wt% and confirms this assumption.

In the third step the sulfates show a thermal stability until 550 °C. A chemically plausible decomposition results in the formation of potassium antimonite^[^ ^56^ ^]^ which typically is synthesized in this temperature range and boron oxide (Δ*m*_calc_ = 14.8 wt%, Δ*m*_obs_ = 14.1 wt%):

$$\begin{array}{cc} & 0.5\;{\text{Sb}}_{2}{\left({\text{SO}}_{4}\right)}_{3}+0.5\;K_{2}{\text{SO}}_{4}+2\;B_{2}O_{3}\\ & \;\to "{\text{KSbO}}_{2}"+2\;B_{2}O_{3}+2.5\;{\text{SO}}_{3} \end{array}$$

The final product, after heating the sample to 1000 °C, could not be analyzed as no residues were left. Besides for the cases of Sb(NO)[B(SO_4_)_2_]_4_ and Sb(NH_4_)[B(SO_4_)_2_]_4_ which presumably result in the formation of Sb_2_O_3_ and B_2_O_3_, it can be assumed that in all other cases in the last decomposition step the respective antimonites are formed as the mass losses fit very well.

Of the remaining compounds, only SbRb[B(SO_4_)_2_]_4_ reveals a similar behavior (Figure S8). We assume that besides Sb_2_(SO_4_)_3_ the intermediate “Rb[B(SO_4_)_2_]”—not known in literature so far—is formed and accordingly follows the same decomposition as the potassium compound.

All remaining TGA curves deviate significantly so that alternative processes have to be considered. TPPXRD studies of SbAg[B(SO_4_)_2_]_4_ and SbTl[B(SO_4_)_2_]_4_ revealed so far unknown diffraction patterns which do not coincide with sulfates and borosulfates known in literature and may be caused by the synthesis of new borosulfates (Figures S9, S10). In general, these deviating decomposition steps indicate that the antimony compounds are more reactive than the bismuth analogues and thus tend to form new compounds. This remains a potential field of study to discover new borosulfates in the future.

## Conclusion

In this contribution we presented a careful analysis of the antimony borosulfates SbX[B(SO_4_)_2_]_4_ (X = Li^+^, Na^+^, K^+^, Rb^+^, Cs^+^, Ag^+^, Tl^+^, NO^+^, NH_4_^+^), within the first modular system in borosulfate chemistry, i.e., M^III^M^I^[B(SO_4_)_2_]_4_ (M^III^ = Bi^3+^, Sb^3+^, Lu^3+^; M^I^ = Li^+^, Na^+^, K^+^, Rb^+^, Cs^+^, NO_2_^+^, H_3_O^+^).^[^ ^25^ ^]^ The titled compounds crystallize in three group–subgroup related space groups *I* $\bar{4}$ (no. 82), *P* $\bar{4}$ (no. 81) and *C*2 (no. 5) where the crystallization not only depends on the incorporated monovalent cation− like observed for the respective bismuth and lutetium compounds−but also on the lone pair effect of antimony. Besides the already known alkali metal compounds, additionally, Ag^+^, Tl^+^, NO^+^, and NH_4_^+^ were incorporated into this structure representing the first compounds in borosulfate chemistry containing thallium and nitrosonium cations. The attempt to synthesize SbX[B(SO_4_)_2_]_4_ (X = Li^+^, Na^+^) resulted in the synthesis of SbX[B_4_O_2_(SO_4_)_6_] (X = Li^+^, Na^+^). The unconventional borosulfates comprising B─O─B bridges crystallize in a new structure type in the space group *Pnma* (no. 62) consisting of unprecedented loop‐branched *achter* double chains. The lone pair effect of antimony overcomes the sterical influence of Li^+^ and Na^+^ that are too small to stabilize the previously mentioned three‐dimensional structure type. ^121^Sb Mössbauer spectra of SbX[B(SO_4_)_2_]_4_ (X = K^+^, Rb^+^, Cs^+^, NO^+^, NH_4_^+^), revealed an extremely negative average isomeric shift of −22 mm·s^−1^ never seen before (Table 2). These results indicate a high *s*‐electron density at the nucleus and hence a very weak coordination behavior of the borosulfate anion. A slightly stronger coordination behavior was observed in SbNa[B_4_O_2_(SO_4_)_6_] where the antimony cation is additionally coordinated by a borate group—compared to the former structure type where a coordination by solely sulfate groups is found—leading to an isomeric shift of −18 mm·s^−1^. DFT calculations confirm these findings by following the linear dependency of the isomer shift to the electron density at the nuclear site (Figure 7). To confirm the non‐centrosymmetric space group and hence validate the structure refinement we performed SHG measurements. Sb(NH_4_)[B(SO_4_)_2_]_4_ showed a non‐linear optical response comparable to potassium dihydrogen phosphate. Conducted IR spectroscopy confirmed the same trend which was already observed in ref. [53] where all spectra only differ in the region around 1350 cm^−1^, the location of symmetric S─O_term_ stretching vibrations of oxygen atoms coordinating the monovalent cations and depended on position on the latter. Furthermore, TGA and TPPXRD experiments were carried out and uncovered a variety of decomposition processes along the series which are not all understood well till date except the one of SbK[B(SO_4_)_2_]_4_. The decompositions of SbTl[B(SO_4_)_2_]_4_ and SbAg[B(SO_4_)_2_]_4_ were followed in further TPPXRD experiments and unveiled unknown diffraction patterns. These findings may indicate the formation of further unknown borosulfates like, e.g., SbAg[B_4_O_2_(SO_4_)_6_] containing both mono and trivalent cations simultaneously and open a potential field of study to find more borosulfates in the future.

## Supporting Information

The authors have cited additional references within the Supporting Information.^[^ ^50^, ^57^, ^58^, ^59^, ^60^, ^61^, ^62^, ^63^, ^64^, ^65^, ^66^, ^67^, ^68^, ^69^, ^70^, ^71^, ^72^, ^73^, ^74^, ^75^, ^76^, ^77^, ^78^, ^79^, ^80^, ^81^, ^82^, ^83^, ^84^, ^85^, ^86^, ^87^ ^]^

## Conflict of Interests

The authors declare no conflict of interest.

## Supporting information



Supporting Information

Supporting Information

## Data Availability

The data that support the findings of this study are available from the corresponding author upon reasonable request.

## References

[1] J. Bruns , H. A. Höppe , M. Daub , H. Hillebrecht , H. Huppertz , Chem. ‐ Eur. J. 2020, 26, 7966–7980, 10.1002/chem.201905449. 31943390 PMC7384169

[2] D. S. Wimmer , M. Seibald , D. Baumann , K. Wurst , H. Huppertz , Chem. ‐ Eur. J. 2023, 29, e202202448, 10.1002/chem.202202448. 36239983 PMC10098606

[3] M. Li , A. Verena‐Mudring , Cryst. Growth Des. 2016, 16, 2441.

[4] S. G. Jantz , M. Dialer , L. Bayarjargal , B. Winkler , L. van Wüllen , F. Pielnhofer , J. Brgoch , R. Weihrich , H. A. Höppe , Adv. Opt. Mater. 2018, 6, 1800497.

[5] A. F. Masters , T. Maschmeyer , Micropor. Mesopor. Mater. 2011, 142, 423–438, 10.1016/j.micromeso.2010.12.026.

[6] M. Daub , H. A. Höppe , H. Hillebrecht , Z. Anorg. Allg. Chem. 2014, 640, 2914–2921, 10.1002/zaac.201400315.

[7] M. Hämmer , L. C. Pasqualini , S. S. Sebastian , H. Huppertz , H. A. Höppe , J. Bruns , Dalton Trans. 2022, 51, 15458–15466, 10.1039/D2DT02344J. 36156042

[8] M. Daub , K. Kazmierczak , P. Gross , H. Höppe , H. Hillebrecht , Inorg. Chem. 2013, 52, 6011. 23656591 10.1021/ic400267s

[9] P. Gross , A. Kirchhain , H. A. Höppe , Angew. Chem. Int. Ed. 2016, 55, 4353. 10.1002/anie.201510612 26924507

[10] P. Netzsch , H. A. Höppe , Eur. J. Inorg. Chem. 2021, 2021, 1065–1070, 10.1002/ejic.202001095.

[11] W. Loewenstein , Am. Mineral. 1954, 39, 92.

[12] L. Pauling , J. Am. Chem. Soc. 1929, 51, 1010–1026, 10.1021/ja01379a006.

[13] J. George , D. Waroquiers , D. Di Stefano , G. Petretto , G. Rignanese , G. Hautier , Angew. Chem. 2020, 132, 7639–7645. Portico. 10.1002/ange.202000829 PMC7217010 32065708

[14] P. Netzsch , M. Hämmer , E. Turgunbajew , T. P. van Swieten , A. Meijerink , H. A. Höppe , M. Suta , Adv. Opt. Mater. 2022, 10, 2200059, 10.1002/adom.202200059.

[15] L. M. Träger , L. C. Pasqualini , H. Huppertz , J. Bruns , M. Suta , Angew. Chem. Int. Ed. 2023, 62, e202309212, 10.1002/anie.202309212. 37548647

[16] M. D. Ward , B. L. Chaloux , M. D. Johannes , A. Epshteyn , Adv. Mater. 2020, 32, 2003667, 10.1002/adma.202003667. 32924200

[17] Y. Li , Z. Zhou , S. Zhao , F. Liang , Q. Ding , J. Sun , Z. Lin , M. Hong , J. Luo , Angew. Chem. Int. Ed. 2021, 60, 11457–11463, 10.1002/anie.202102107. 33686736

[18] Z. Li , W. Jin , F. Zhang , Z. Yang , S. Pan , ACS Cent. Sci. 2022, 8, 1557–1564, 10.1021/acscentsci.2c00832. 36439311 PMC9686211

[19] P. Netzsch , P. Gross , H. Takahashi , H. A. Höppe , Inorg. Chem. 2018, 57, 8530–8539, 10.1021/acs.inorgchem.8b01234. 29957944

[20] P. Netzsch , M. Hämmer , P. Gross , H. Bariss , T. Block , L. Heletta , R. Pöttgen , J. Bruns , H. Huppertz , H. A. Höppe , Dalton Trans. 2019, 48, 4387–4397, 10.1039/C9DT00445A. 30864591

[21] D. van Gerven , S. Sutorius , J. Bruns , M. S. Wickleder , ChemistryOpen 2022, 11, e202200122. 35856862 10.1002/open.202200122 PMC9630045

[22] P. E. Lippens , Solid State Commun. 2000, 113, 399–403, 10.1016/S0038-1098(99)00501-3.

[23] J. D. Donaldson , M. J. Tricker , B. W. Dale , J. Chem. Soc. Dalton Trans. 1972, 8, 893.

[24] CSD Deposition numbers 2410212 (for SbLi[B(SO_4_)_2_]_4_), 2410213 (for SbNa[B(SO_4_)_2_]_4_), 2410216 (for SbCs[B(SO_4_)_2_]_4_), 2491527 (for Sb(NH_4_)[B(SO_4_)_2_]_4_), 2491528 (for Sb(NO)[B(SO_4_)_2_]_4_), 2410214 (for SbK[B(SO_4_)_2_]_4_), 2410215 (for SbRb[B(SO_4_)_2_]_4_), 2491529 (for SbAg[B(SO_4_)_2_]_4_), 2491530 (for SbTl[B(SO_4_)_2_]_4_) and 2491664 (for SbNa[B_4_O_2_(SO_4_)_6_]) contain the supplementary crystallographic data for this paper. These data are provided free of charge by the joint Cambridge Crystallographic Data Centre and Fachinformationszentrum Karlsruhe Access Structures service.

[25] E. Turgunbajew , H. A. Höppe , Angew. Chem. Int. Ed. 2025, 64, e202424952. 10.1002/anie.202424952 39936233

[26] R. D. Shannon , Acta Crystallogr. A 1976, 32, 751–767, 10.1107/S0567739476001551.

[27] L. C. Pasqualini , O. Janka , S. Olthof , H. Huppertz , K. R. Liedl , R. Pöttgen , M. Podewitz , J. Bruns , Chem. ‐ Eur. J. 2020, 26, 17405–17415, 10.1002/chem.202002221. 32557937 PMC7820960

[28] R. Hübenthal , MAPLE: Programm for Calculation of the Madelung Part of Lattice Energy, Universität Gießen, Gießen, 1993.

[29] R. Hoppe , Angew. Chem. Int. Ed. 1966, 5, 95–106, 10.1002/anie.196600951.

[30] R. Hoppe , Angew. Chem. 1966, 78, 52–63, 10.1002/ange.19660780106.

[31] R. Hoppe , Angew. Chem. Int. Ed. 1970, 9, 25–34, 10.1002/anie.197000251.

[32] R. Hoppe , Z. Kristallogr. 1979, 150, 23–52, 10.1524/zkri.1979.150.1-4.23.

[33] A. Walsh , D. J. Payne , R. G. Egdell , G. W. Watson , Chem. Soc. Rev. 2011, 40, 4455, 10.1039/c1cs15098g. 21666920

[34] D. J. Payne , R. G. Egdell , A. Walsh , G. W. Watson , J. Guo , P.‐A. Glans , T. Learmonth , K. E. Smith , Phys. Rev. Lett. 2006, 96, 157403, 10.1103/PhysRevLett.96.157403. 16712195

[35] M. Hämmer , J. Brgoch , P. Netzsch , H. A. Höppe , Inorg. Chem. 2022, 61, 4102–4113, 10.1021/acs.inorgchem.1c03893. 35192329

[36] T. Balić Žunić , E. Makovicky , Acta Crystallogr. B 1996, 52, 78.

[37] E. Makovicky , T. B. Žunić , Acta Crystallogr. B 1998, 54, 766–773, 10.1107/S0108768198003905.

[38] E. Turgunbajew , G. Buchner , H. A. Höppe , *unpublished results*.

[39] I. D. Brown , Acta Crystallogr. B 1988, 44, 545–553, 10.1107/S0108768188007712.

[40] A. L. Spek , *PLATON, A Multipurpose Crystallographic Tool*, University Utrecht, Utrecht, 2002.

[41] A. L. Spek , J. Appl. Crystallogr. 2003, 36, 7–13, 10.1107/S0021889802022112.

[42] A. L. Spek , Acta Crystallogr. D 2009, 65, 148–155, 10.1107/S090744490804362X. 19171970 PMC2631630

[43] F. Liebau , Structural Chemistry of Silicates. Structure, Bonding, and Classification, Springer Heidelberg, 1985, 10.1007/978-3-642-50076-3.

[44] P. Netzsch , P. Gross , H. Takahashi , S. Lotfi , J. Brgoch , H. A. Höppe , Eur. J. Inorg. Chem. 2019, 2019, 3975–3981, 10.1002/ejic.201900838.

[45] M. Hämmer , H. A. Höppe , Z. Anorg. Allg. Chem. 2022, 648, e202200197.

[46] N. N. Greenwood , T. C. Gibb , Mössbauer Spectroscopy, Chapman and Hall Ltd, London, 1971.

[47] J. G. Stevens , in Chemical Mössbauer Spectroscopy (Ed.: R. H. Herber ), Plenum Press New York, 1984, pp. 319–342.

[48] P. E. Lippens , J. C. Jumas , J. Olivier‐Fourcade , Hyperfine Int 2002, 141, 303–308.

[49] Y. Kajitani , M. Takahashi , M. Takeda , Int. J. Inorg. Mater. 2001, 3, 337–340, 10.1016/S1466-6049(01)00033-2.

[50] T. Birchall , B. D. Valle , J. Chem. Soc. D 1970, 675–676, 10.1039/C29700000675.

[51] S. L. Ruby , G. K. Shenoy in Mössbauer Isomer Shifts (Eds.: G. K. Shenoy , F. E. Wagner ), North‐Holland Publishing Company, Amsterdam, 1978, pp. 617–659.

[52] J. G. Ballard , T. Birchall , J. B. Milne , W. D. Moffett , Can. J. Chem. 1974, 52, 2375–2379, 10.1139/v74-344.

[53] E. Turgunbajew , M. Hämmer , L. Bayarjargal , F. Pielnhofer , H. A. Höppe , Chem. ‐ Eur. J. 2025, 31, e02439, 10.1002/chem.202502439. 41047760 PMC12587024

[54] J. Bruns , M. Podewitz , M. Schauperl , K. R. Liedl , O. Janka , R. Pöttgen , H. Huppertz , Eur. J. Inorg. Chem. 2017, 2017, 3981–3989, 10.1002/ejic.201700360.

[55] P. Netzsch , F. Pielnhofer , H. A. Höppe , Inorg. Chem. 2020, 59, 15180–15188, 10.1021/acs.inorgchem.0c02156. 33001636

[56] C. Hirschle , C. Röhr , Z. Anorg. Allg. Chem. 2000, 626, 1305–1312, 10.1002/(SICI)1521-3749(200006)626:6<1305::AID-ZAAC1305>3.0.CO;2-L.

[57] D. H. Moseley , R. Juneja , L. L. Daemen , I. Sergueev , R. Steinbrügge , O. Leupold , Y. Cheng , V. R. Cooper , L. Lindsay , M. K. Kidder , M. E. Manley , R. P. Hermann , Inorg. Chem. 2023, 62, 16464–16474, 10.1021/acs.inorgchem.3c02189. 37747902

[58] L. Stievano , F. E. Wagner , H. W. Zanthoff , S. Calogero , Hyperfine Int 2002, 141‐142, 397–402, 10.1023/A:1021200808596.

[59] D. J. Stewart , O. Knop , C. Ayasse , F. W. D. Woodhams , Can. J. Chem. 1972, 50, 690–700, 10.1139/v72-106.

[60] G. G. Long , J. G. Stevens , L. H. Bowen , Inorg. Nucl. Chem. Lett. 1969, 5, 799–804, 10.1016/0020-1650(69)80061-9.

[61] R. A. Brand, *WinNormaos for Igor7 (version for Igor 7.010 or above: 01/03/2020)*, Universität Duisburg, Duisburg (Germany), 2020.

[62] *CorelDRAW Graphics Suite p. 2017 (version 19.0.0.328)*, Corel Corporation, Ottawa, Ontario (Canada), 2017.

[63] J. W. Zwanziger , J. Phys.: Condens. Matter 2009, 21, 195501. 21825488 10.1088/0953-8984/21/19/195501

[64] G. Kresse , J. Furthmüller , Phys. Rev. B 1996, 54, 11169–11186, 10.1103/PhysRevB.54.11169. 9984901

[65] G. Kresse , J. Furthmüller , Comput. Mat. Sci. 1996, 6, 15–50, 10.1016/0927-0256(96)00008-0.

[66] G. Kresse , J. Hafner , Phys. Rev. B 1993, 47, 558–561, 10.1103/PhysRevB.47.558. 10004490

[67] P. E. Blöchl , Phys. Rev. B 1994, 50, 17953. 10.1103/physrevb.50.17953 9976227

[68] G. Kresse , D. Joubert , Phys. Rev. B 1999, 59, 1758–1775, 10.1103/PhysRevB.59.1758.

[69] H. J. Monkhorst , J. D. Pack , Phys. Rev. B 1976, 13, 5188–5192, 10.1103/PhysRevB.13.5188.

[70] F. Tran , P. Blaha , Phys. Rev. Lett. 2009, 102, 226401, 10.1103/PhysRevLett.102.226401. 19658882

[71] T. W. D. Farley , W. Hayes , S. Hull , M. T. Hutchings , M. Vrtis , J. Phys.: Condens. Matter 1991, 3, 4761.

[72] E. Zintl , A. Harder , B. Dauth , Z. Elektrochem. 1934, 40, 588.

[73] P. Touzain , F. Brisse , M. Caillet , Can. J. Chem. 1970, 48, 3358–3361, 10.1139/v70-564.

[74] A. Helms , W. Klemm , Z. Anorg. Allg. Chem. 1939, 242, 33–40, 10.1002/zaac.19392420103.

[75] K.‐R. Tsai , P. M. Harris , E. N. Lassettre , J. Phys. Chem. B 1956, 60, 338–344, 10.1021/j150537a022.

[76] H. Sabrowsky , Z. Anorg. Allg. Chem. 1971, 381, 266–279, 10.1002/zaac.19713810305.

[77] P. Norby , R. Dinnebier , A. N. Fitch , Inorg. Chem. 2002, 41, 3628–3637, 10.1021/ic0111177. 12099865

[78] A. E. Whitten , B. Dittrich , M. A. Spackman , P. Turner , T. C. Brown , Dalton Trans. 2004, 23–29, 10.1039/b312550e. 15356737

[79] G. E. Gurr , P. W. Montgomery , C. D. Knutson , B. T. Gorres , Acta Crystallogr. B 1970, 26, 906–915, 10.1107/S0567740870003369.

[80] R. Pascard , C. Pascard‐Billy , Acta Crystallogr. 1965, 18, 830–834, 10.1107/S0365110X65002049.

[81] J. Horakh , H. Borrmann , A. Simon , Chem. ‐ Eur. J. 1995, 1, 389–393, 10.1002/chem.19950010610.

[82] R. Boese , N. Niederprüm , D. Bläser , A. Maulitz , M. Y. Antipin , P. R. Mallinson , J. Phys. Chem. B 1997, 101, 5794–5799, 10.1021/jp970580v.

[83] J. P. Devort , J. M. Friedt , Chem. Phys. Lett. 1975, 35, 423–425, 10.1016/0009-2614(75)85635-1.

[84] J. G. Ballard , T. Birchall , D. R. Slim , J. Chem. Soc. Dalton Trans. 1977, 15, 1469.

[85] R. A. Pruitt , S. W. Marshall , C. M. O'Donnell , Phys. Rev. B 1970, 2, 2383–2390, 10.1103/PhysRevB.2.2383.

[86] S. L. Ruby , G. M. Kalvius , G. B. Beard , R. E. Snyder , Phys. Rev. B 1967, 159, 239–245, 10.1103/PhysRev.159.239.

[87] P. R. Mercier , J. Douglade , J. Bernard , Acta Crystallogr. B 1976, 32, 2787–2791, 10.1107/S0567740876008881.

