# Supplementary material for: Mössbauer Spectroscopy on Antimony Borosulfates Reveals Weak Coordination Behavior
Source: Angew Chem Int Ed Engl. 2025 Dec 12;65(4):e21198. doi: 10.1002/anie.202521198 (PMC12828460; doi:10.1002/anie.202521198)
Supplement: Supplementary file 1 — Supporting Information [file ANIE-65-e21198-s002.pdf]

Angewandte Chemie

Supporting Information

Mössbauer Spectroscopy on Antimony Borosulfates Reveals Weak Coordination Behaviour

Erich Turgunbajew,[a] Gwendolyn Buchner,[a] Aylin Koldemir,[b] Theresa Block,[b] Rainer Pöttgen,[b] David Hemker,[c] Richard Dronskowski,[c] and Henning A. Höppe*[a]

Experimental Section

Synthesis

$SbM^I[B(SO_4)_2]_4$ (M^I = Li^+, Na^+, K^+, Rb^+, Cs^+, Tl^+, Ag^+, NH_4^+, NO^+) were synthesised using Sb_2O_3 (0.13 mmol), the respective metal sulfate $M^I_2(SO_4)$ (M^I = Li^+, Na^+, K^+, Cs^+, Tl^+, Ag^+, NH_4^+) (0.26 mmol) and B_2O_3 (1.04 mmol). Regarding $SbRb[B(SO_4)_2]_4$, the metal carbonate Rb_2CO_3 (0.22 mmol) was used. The educts were ground and transferred into silica ampoules (length: 12 cm, diameter: 1 cm, wall thickness: 0.1 cm) together with 1 mL of oleum 65% (VWR, 65% SO_3). In the case of $Sb(NO)[B(SO_4)_2]_4$ prior to the addition of oleum 0.2 mL $NOHSO_4$ solution (Sigma-Aldrich, $NOHSO_4$ in sulfuric acid 40 wt%) was added. Afterwards, the ampoules were torch sealed, placed in a muffle furnace and without further aging the following temperature program was applied: heating to 180°C with 50°K/h, holding the temperature for 24 h, and cooling to room temperature with 6°K/h. After decantation of the excess oleum, the ampoules were frozen with liquid nitrogen and eventually opened. (**Caution**: During and even after the reaction, the ampules are under remarkable pressure and must therefore be handled with great care, e.g., they must be cooled with liquid nitrogen prior to opening.) The moisture sensitive products were washed with 2 mL anhydrous acetonitrile using a setup under nitrogen atmosphere (Acros, 99.9%, extra dry) and accordingly transferred into an argon filled glovebox.

Single crystal X-ray diffraction

Immediately after opening the ampoule, single crystals were transferred from the mother liquor into perfluorinated polyether. Suitable crystals were picked under a polarization microscope, mounted onto a MicroMount (MiTeGen) and directly transferred into a cold nitrogen gas stream (Oxford Cryostream Plus). Single Crystal X-ray Diffraction data was collected on a Bruker D8 Venture under $Mo_{K\alpha}$ radiation (0.71073 Å) equipped with a PHOTON-100 detector. Absorption correction was performed via the multi-scan method. [1] The crystal structures were solved by Direct Methods using the SHELXS program [2] and refined by full-matrix least-squares technique with the SHELXTL program. [3]

(Temperature Programmed) Powder X-ray diffraction

The samples were ground and filled into a Hilgenberg glass capillary (outer diameter 0.3 mm, wall thickness 0.01 mm). The PXRD patterns were recorded with a D8 Venture in transmission geometry using Cu-K_α radiation (1.54184 Å), a 1D LynxEye detector and a nickel filter to suppress K_β radiation. Temperature programmed PXRD diagrams were recorded on the same device using an oven attachment.

Infrared Spectroscopy

The infrared spectra were recorded using an EQUINOX 55 FT-IR spectrometer (Bruker) equipped with a platinum ATR unit in the range of 4000-400 cm^{-1} and a resolution of 4 cm^{-1} with 32 scans per measurement.

Thermogravimetric Analysis

The thermogravimetric analyses were performed in alumina crucibles, under nitrogen atmosphere and a heating rate of 5°C/min using a STA 409 PC Luxx.

^{121}Sb Mössbauer Spectroscopy

The ^{121}Sb Mössbauer spectroscopic investigation of the different borosulfate samples was performed in usual transmission geometry. A $Ba^{121m}SnO^3$ source was used for the measurements. The samples were enclosed in thin-walled PMMA containers under argon and set to 78 K inside a liquid nitrogen bath cryostat, while the source was kept at room temperature. A sample of binary $Sb^{III}Sb^{V}O_4$ [4–8] was measured for comparison. Fitting of the spectra was done by using the WinNormos for Igor7 software package [9] and the graphical editing was performed with the program CorelDRAW2017. [10]

DFT calculation

Following the procedure of Zwanziger [11] the ^{121}Sb Mössbauer isomer shift was estimated by correlating the electron density at the Sb atomic positions to the Mössbauer isomer shift. The electron densities of several reference compounds, as well as the one of $Sb(NH_4)[B(SO_4)_2]$, were calculated with VASP, [12–14] using PAW pseudopotential [15,16] on properly converged Monkhorst–Pack *k*-point grids [17] and plane-wave energy cutoffs more than 520 eV. A modified version of the exchange potential proposed by Becke and Johnson [18] was used, as it promises accurate wave functions and, therefore, densities, at a relatively low prize. The results are summarized in Table S11.Fehler: Verweis nicht gefunden

Table S1. Crystal data, structural refinement and CSD numbers of $SbX[B(SO_4)_2]_4$ (X = Li^+, Na^+, Ag^+, Cs^+). Standard deviation is given in parentheses.

	**SbLi[B(SO4)$_2$]$_4$**	**SbNa[B(SO4)$_2$]$_4$**	**SbAg[B(SO4)$_2$]$_4$**	**SbCs[B(SO$_4$)$_2$]$_4$**
temperature / K	200(2)	200(2)	200(2)	200(2)
molar mass / g·mol^{-1}	940.44	956.4946	1041.37	1066.41
crystal system	tetragonal	tetragonal	tetragonal	tetragonal
crystal size / mm^3	0.14 x 0.08 x 0.07	0.21 x 0.09 x 0.07	0.13 x 0.07 x 0.06	0.12 x 0.07 x 0.05
space group	$I\bar{4}$ (No. 82)	$I\bar{4}$ (No. 82)	$P\bar{4}$ (No. 81)	$P\bar{4}$ (No. 81)
a / Å	11.7805(3)	11.7889(6)	11.7994(4)	11.9163(10)
c / Å	8.1027(3)	8.1190(6)	8.1323(3)	8.0845(1)
volume / Å^3	1124.49(7)	1128.36(14)	1132.23(9)	1147.98(3)
Z	2	2	2	2
ρ_{calc} / g·cm^{-3}	2.78	2.81	3.05	3.08
absorption coefficient μ / mm^{-1}	2.1	2.1	2.9	3.6
F(000) / e	916	932	1004	1020
Flack parameter	0.001(3)	0.004(9)	0.015	0.007(8)
radiation wavelength λ / Å	0.71073	0.71073	0.71073	0.71073
absorption correction	multi-scan	multi-scan	multi-scan	multi-scan
transmission factor (min./max.)	0.676 / 0.748	0.676 / 0.748	0.639 / 0.715	0.650 / 0.748
Index range *h*/*k*/*l*	±21 | ±21 | ±14	-14/19 |-18/19 | ±13	±19 | -16/19 | ±13	±19 | -19/18 | ±13
theta range / °	2.445-34.968	2.443-34.997	2.441-34.967	2.417-34.987
reflections collected	24708	14084	51903	29494
independent reflections	3485	2472	4968	5036
observed reflections	3390	2438	4573	4836
refined parameters /restraints	109 / 1	112 / 1	213 / 1	210 / 0
R_{int}	0.047	0.042	0.099	0.055
R_σ	0.032	0.034	0.042	0.041
R_1 (all data)	0.018	0.025	0.028	0.027
wR_2 (all data)	0.045	0.058	0.055	0.061
Goof	0.993	1.042	1.044	1.042
residual electron density (min./max.) / e$^-$ Å^{-3}	-0.80 / 0.38	-2.10 / 0.83	-1.54 / 0.83	-2.02 / 0.58
CSD number	2410212	2410213	2491529	2410216

Table S2. Crystal data, structural refinement and CSD numbers of $SbX[B(SO_4)_2]_4$ (X = NH_4^+, NO^+, K^+, Rb^+, Tl^+). Standard deviation is given in parentheses.

	**$Sb(NH_4)[B(SO_4)_2]_4$**	**$Sb(NO)[B(SO_4)_2]_4$**	**$SbK[B(SO4)_2]_4$**	**$SbRb[B(SO4)_2]_4$**	**$SbTl[B(SO4)_2]_4$**
temperature/ K	250(2)	200(2)	200(2)	200(2)	200(2)
molar mass / g·mol^{-1}	951.51	963.51	972.57	1018.94	1137.84
crystal system	monoclinic	monoclinic	monoclinic	monoclinic	monoclinic
crystal size / mm^3	0.17 x 0.14 x 0.08	0.09 x 0.05 x 0.03	0.09 x 0.07 x 0.06	0.08 x 0.07 x 0.05	0.15 x 0.08 x 0.07
space group	*C*2 (No. 5)	*C*2 (No. 5)	*C*2 (No. 5)	*C*2 (No. 5)	*C*2 (No. 5)
a / Å	16.6782(7)	16.6672(5)	16.619(3)	16.6673(5)	16.689(2)
b / Å	8.1140(3)	8.1228(2)	8.1217(16)	8.1124(2)	8.1093(8)
c / Å	11.8248(9)	11.7741(4)	11.723(2)	11.8308(6)	11.8595(14)
β / °	134.79(10)	134.84(10)	134.55(5)	134.73(10)	134.72(1)
volume / Å^3	1135.60(11)	1130.21(6)	1127.6(4)	1136.45(7)	1140.5(2)
Z	2	2	2	2	2
ρ_{calc} / g·cm^{-3}	2.78	2.83	2.86	2.98	3.31
absorption coefficient μ / mm^{-1}	2.1	2.1	2.3	4.2	9.1
F(000) / e	932	940	948	984	1072
Flack parameter	0.05(3)	0.05(3)	0.001(3)	0.032(10)	0.028(7)
radiation wavelength λ / Å	0.71073	0.71073	0.71073	0.71073	0.71073
absorption correction	multi-scan	multi-scan	multi-scan	multi-scan	multi-scan
transmission factor (min./max.)	0.695 / 0.751	0.619 / 0.701	0.620 / 0.703	0.655 / 0.749	0.497 / 0.687
Index range *h*/*k*/*l*	±26 | -11/12 | ±18	±26 | ±13 | ±18	±26 | ±13 | ±18	±26 | ±13 | ±19	±28 | ±13 | ±20
theta range / °	2.442-32.493	2.440-34.967	2.438-34.997	2.423-34.998	2.417-37.497
reflections collected	25941	30251	29182	33726	21250
independent reflections	4053	4928	4924	4968	3810
observed reflections	4018	4864	4886	4785	3759
refined parameters /restraints	225 / 13	215 / 4	213 / 1	213 / 1	213 / 1
R_{int}	0.033	0.058	0.039	0.060	0.038
R_σ	0.026	0.039	0.032	0.043	0.047
R_1 (all data)	0.039	0.039	0.017	0.029	0.030
wR_2 (all data)	0.102	0.088	0.041	0.061	0.087
Goof	1.361	1.270	1.078	1.102	1.138
residual electron density (min./max.) / e$^-$ Å^{-3}	-1.68 / 1.14	-1.49 / 1.43	-0.46 / 0.47	-1.43 / 0.96	-1.80 / 2.91
CSD number	2491527	2491528	2410214	2410215	2491530

Table S3. Crystal data, structural refinement and CSD number of $SbNa[B_4O_2(SO_4)_6]_4$. Standard deviation is given in parentheses.

	$SbNa[B_4O_2(SO_4)_6]$
temperature/ K	100(2)
molar mass / g·mol^{-1}	796.34
crystal system	orthorhombic
crystal size / mm^3	0.24 x 0.14 x 0.13
space group	*Pnma* (No. 62)
a / Å	13.8021(7)
b / Å	11.6319(5)
c / Å	10.9307(5)
volume / Å^3	1754.87(13)
Z	2
ρ_{calc} / g·cm^{-3}	3.01
absorption coefficient μ / mm^{-1}	2.5
F(000) / e	1544
radiation wavelength λ / Å	0.71073
absorption correction	multi-scan
transmission factor (min./max.)	0.646 / 0.752
Index range *h*/*k*/*l*	±22 | ±18 | ±17
theta range / °	2.377-34.998
reflections collected	57739
independent reflections	4011
observed reflections	3825
refined parameters /restraints	187 / 0
R_{int}	0.073
R_σ	0.031
R_1 (all data)	0.024
wR_2 (all data)	0.053
Goof	1.048
residual electron density (min./max.) / e$^-$ Å^{-3}	-0.79 / 1.22
CSD number	2491664

Table S4. Calculated MAPLE values of $SbX[B(SO_4)_2]_4$ (X = Li^+, Na^+, K^+, Rb^+, Cs^+, Ag^+, Tl^+, NO^+, NH_4^+) compared to the respective sum of the MAPLE values of the binary oxides and NH_3.

used compounds	MAPLE / $kJ \cdot mol^{-1}$
Li_2O [19]	3506
Na_2O [20]	2907
K_2O [21]	2511
Rb_2O [22]	2397
Cs_2O [23]	2226
Tl_2O [24]	2353
Ag_2O [25]	3012
Sb_2O_3 [26]	14976
B_2O_3 [27]	21924
SO_3 [28]	29930
N_2O_3 [29]	16536
NH_3 [30]	10054
0.5 Sb_2O_3 + 2 B_2O_3 + 8 SO_3 + 0.5 Li_2O	292531
$SbLi[B(SO_4)_2]_4$	293224
Δ = 0.23 %	
0.5 Sb_2O_3 + 2 B_2O_3 + 8 SO_3 + 0.5 Na_2O	292232
$SbNa[B(SO_4)_2]_4$	293081
Δ = 0.29 %	
0.5 Sb_2O_3 + 2 B_2O_3 + 8 SO_3 + 0.5 K_2O	292034
$SbK[B(SO_4)_2]_4$	292874
Δ = 0.28 %	
0.5 Sb_2O_3 + 2 B_2O_3 + 8 SO_3 + 0.5 Rb_2O	291977
$SbRb[B(SO_4)_2]_4$	291562
Δ = 0.14 %	
0.5 Sb_2O_3 + 2 B_2O_3 + 8 SO_3 + 0.5 Cs_2O	291891
$SbCs[B(SO_4)_2]_4$	292786
Δ = 0.30 %	
0.5 Sb_2O_3 + 2 B_2O_3 + 8 SO_3 + 0.5 Tl_2O	291955
$SbTl[B(SO_4)_2]_4$	292526
Δ = 0.19 %	
0.5 Sb_2O_3 + 2 B_2O_3 + 8 SO_3 + 0.5 Ag_2O	291986
$SbAg[B(SO_4)_2]_4$	292786

$\Delta = 0.31$ %	
0.5 Sb_2O_3 + 2 B_2O_3 + 8 SO_3 + 0.5 N_2O_3<br>$Sb(NO)[B(SO_4)_2]_4$	299047<br>296567
$\Delta = 0.84$ %	
0.5 Sb_2O_3 + 2 B_2O_3 + 8 SO_3 + NH_3 + 0.5 H_2O<br>$Sb(NH_4)[B(SO_4)_2]_4$	302197<br>298478
$\Delta = 1.24$ %	
0.5 Sb_2O_3 + 2 B_2O_3 + 8 SO_3 + 0.5 N_2O_3<br>$SbNa[B_4O_2(SO_4)_6]$	232372<br>232724
$\Delta = 0.15$ %	

Table S5. Centroid deviation R_c, coordination number and eccentricity of $SbX[B(SO_4)_2]_4$ (X = Li^+, Na^+, K^+, Rb^+, Cs^+, Ag^+, Tl^+, NO^+, NH_4^+) in comparison to $Sb_2(SO_4)_3$.

compound	r_{ion}[8] (M^I) / pm	R_c / pm	CN (Sb^{3+})	eccentricity ε	reference
$Sb(NH_4)[B(SO_4)_2]_4$	154	12	8	0.049	this work
$Sb(NO)[B(SO_4)_2]_4$		11	8	0.045	this work
$SbLi[B(SO_4)_2]_4$	99	0.018	8	0	this work
$SbNa[B(SO_4)_2]_4$	118	0.021	8	0	this work
$SbK[B(SO_4)_2]_4$	137	18	8	0.074	this work
$SbRb[B(SO_4)_2]_4$	151	19	8	0.078	this work
$SbCs[B(SO_4)_2]_4$	174	0.049	8	0	this work
$SbAg[B(SO_4)_2]_4$	128	25	8	0.102	this work
$SbTl[B(SO_4)_2]_4$	159	19	8	0.078	this work
$SbNa[B_4O_2(SO_4)_6]$	118	49	6	0.197	this work
$Sb_2(SO_4)_3$ (I)		39	5	0.162	[31]
$Sb_2(SO_4)_3$ (II)		51	4	0.209	[31]

Table S6. Selected bond parameters of $SbX[B(SO_4)_2]_4$ (X = NO^+, NH_4^+, Li^+, Na^+, K^+, Rb^+, Cs^+, Tl^+, Ag^+).

	**Sb – O**	**S – O$_{br}$**	**S – O$_{term}$(Sb^{3+})**	**S – O$_{term}$ (X$^+$)**	**B – O**
Σ r_{ion}	2.38		1.47		1.46
$Sb(NH_4)[B(SO_4)_2]_4$	2.312(7) – 2.525(7)	1.515(6) – 1.539(6)	1.450(7) – 1.467(7)	1.407(7) – 1.416(7)	1.457(10) – 1.479(11)
$Sb(NO)[B(SO_4)_2]_4$	2.305(4) – 2.526(4)	1.517(4) – 1.537(4)	1.438(4) – 1.460(4)	1.408(4) – 1.426(4)	1.460(6) – 1.475(6)
$SbLi[B(SO_4)_2]_4$	2.350(16) – 2.419(17)	1.511(16) – 1.5367(16)	1.451(18) – 1.453(18)	1.416(18) – 1.419(19)	1.458(3) – 1.477(3)
$SbNa[B(SO_4)_2]_4$	2.335(2) – 2.444(18)	1.518(18) – 1.536(19)	1.447(2) – 1.456(2)	1.410(2) – 1.417(2)	1.459(3) – 1.475(3)
$SbK[B(SO_4)_2]_4$	2.293(16) – 2.586(15)	1.518(15) – 1.546(16)	1.439(16) – 1.461(16)	1.409(17) – 1.425(16)	1.452(2) – 1.478(3)
$SbRb[B(SO_4)_2]_4$	2.293(3) – 2.597(3)	1.511(3) – 1.542(3)	1.435(3) – 1.459(3)	1.410(3) – 1.421(3)	1.455(5) – 1.471(5)
$SbCs[B(SO_4)_2]_4$	2.358(3) – 2.438(3)	1.515(3) – 1.544(3)	1.447(3) – 1.452(3)	1.418(3) – 1.424(3)	1.454(5) – 1.479(5)
$SbAg[B(SO_4)_2]_4$	2.264(3) – 2.479(11)	1.518(3) – 1.541(3)	1.434(3) – 1.467(3)	1.404(4) – 1.423(3)	1.453(5) – 1.471(5)
$SbTl[B(SO_4)_2]_4$	2.291(4) – 2.592(5)	1.514(4) – 1.548(4)	1.441(5) – 1.463(4)	1.410(5) – 1.420(4)	1.451(7) – 1.471(7)

Table S7. Selected bond parameters of $SbNa[B_4O_2(SO_4)_6]$.

	**Σ r_{ion}**	**$SbNa[B_4O_2(SO_4)_6]$**
Sb - O	2.38	2.063(15) – 2.668(14)
Na - O	2.52	2.287(18) – 2.533(14)
S – O$_{br}$		1.499(11) – 1.538(11)
S – O$_{term}$(Sb^{3+})	1.47	1.431(15) – 1.481(11)
S – O$_{term}$ (X$^+$)		1.407(15) – 1.429(12)
B – O (B^{3+})	1.46	1.391(16) – 1.424(16)
B – O (S^{6+})		1.476(18) – 1.539(18)

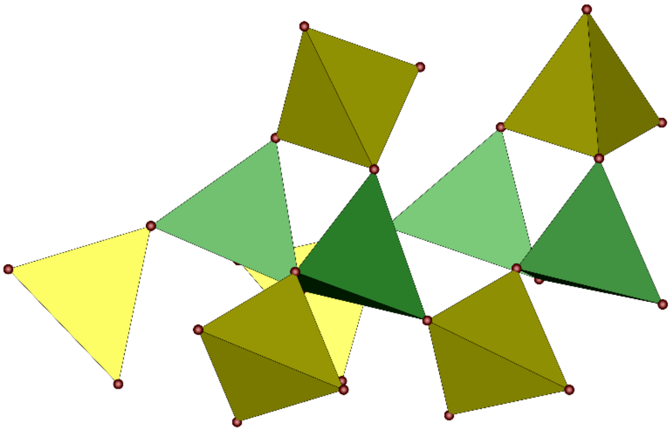

Figure S1. Fundamental building unit of $SbNa[B_4O_2(SO_4)_6]$ comprising the $[B_4O_2(SO_4)_6]^{4-}$ anion.

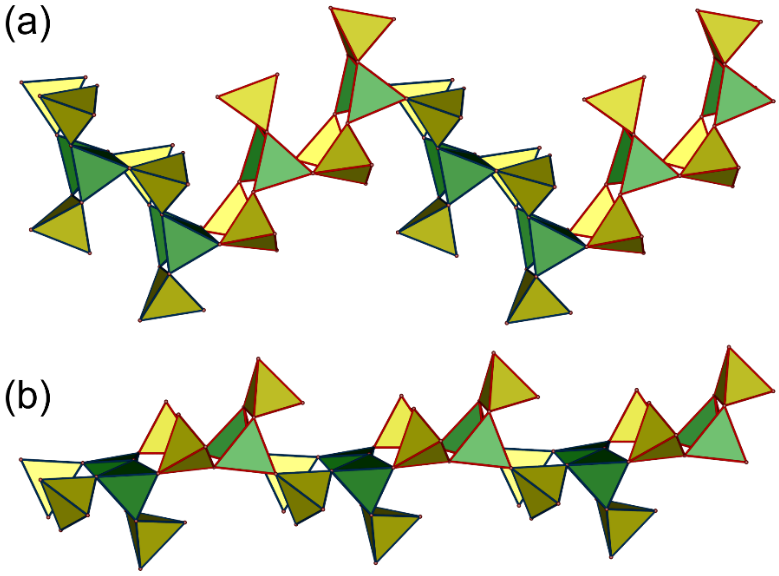

Figure S2. Sections of the anionic chains in (a) $SbNa[B_4O_2(SO_4)_6]$ and (b) $Cd[B_2O(SO_4)_3]$. All $[B_2O(SO_4)_3]^{2-}$ units pointing upwards are marked with red lines and all units pointing downwards are marked in blue. borate in green; sulfate in yellow.

Table S8. ECoN derived by MAPLE calculations for Sb atoms in $SbNa[B_4O_2(SO_4)_6]$

Atom	x	y	z	Distance / pm	Econ(1)	Econ(3)
Central atom						
Sb1	0.8970	¾	0.9449			
Ligand						
O12	0.7501	¾	0.9796	206.251	1.266	1.332
O32	0.8549	0.6363	0.8003	214.141	1.044	1.113
O32	0.8549	0.8637	0.8003	214.141	1.044	1.113
O42	0.8746	0.5684	1.0487	241.716	0.375	0.428
O42	0.8746	0.9316	1.0487	241.716	0.375	0.428
OB2	1.0459	¾	0.7894	266.773	0.076	0.096
Next ligand						
O22	0.9473	¾	1.2129	301.090	0.002	0.003

Table S9. Deviations from tetrahedral symmetry based on the method of Balić-Žunić and Mackovicky for $SbX[B(SO_4)_2]_4$ (X = NO^+, NH_4^+, Li^+, Na^+, K^+, Rb^+, Cs^+, Tl^+, Ag^+). Values are given in %.

M^+	SO_4(1)	SO_4(2)	SO_4(3)	SO_4(4)	BO_4(1)	BO_4(2)
NO^+	0.33	0.15	0.26	0.14	0.28	0.35
NH_4^+	0.26	0.23	0.10	0.15	0.26	0.29
Li^+	0.27	0.16	–	–	0.33	–
Na^+	0.31	0.14	–	–	0.32	–
K^+	0.36	0.25	0.14	0.18	0.26	0.36
Rb^+	0.32	0.25	0.14	0.15	0.26	0.36
Cs^+	0.22	0.29	0.16	0.13	0.30	0.25
Tl^+	0.31	0.22	0.14	0.17	0.29	0.39
Ag^+	0.21	0.12	0.27	0.15	0.28	0.33

Table S10. ECoN derived by MAPLE calculations for Sb atoms in $SbK[B(SO_4)_2]_4$.

Atom	x	y	z	Distance / pm	EcoN(1)	EcoN(3)
Central atom						
Sb1	0	0	0			
Ligand						
O32	0.1517	-0.0767	0.2602	229.28	1.154	1.166
O32	-0.1517	-0.0767	-0.2602	229.28	1.154	1.166
O22	0.0745	-0.2170	-0.0278	230.38	1.126	1.138
O22	-0.0745	-0.2170	0.0278	230.38	1.126	1.138
O42	-0.1209	0.1038	0.0260	238.75	0.912	0.924
O42	0.1209	0.1038	-0.0260	238.75	0.912	0.924
O12	-0.1085	0.2501	-0.1893	258.57	0.467	0.477
O12	0.1085	0.2501	0.1893	258.57	0.467	0.477
Next ligand						
O21	-0.0079	-0.2024	-0.3016	381.44	0	0

Table S11: Valence electron densities, calculated using density functional theory, and experimental ^{121}Sb Mössbauer isomer shifts for a set of reference compounds.

compound	Valence Density (*e*)	Isomer Shift (mm s^{-1})	Reference
$NaSbF_6$	37.23	2.9	[32]
Sb_2O_5	73.10	0.1	[5]
$SbCl_3F_2$	107.20	-1.7	[33]
AlSb	141.80	-7.8	[34]
SbF_3	231.85	-14.7	[35]

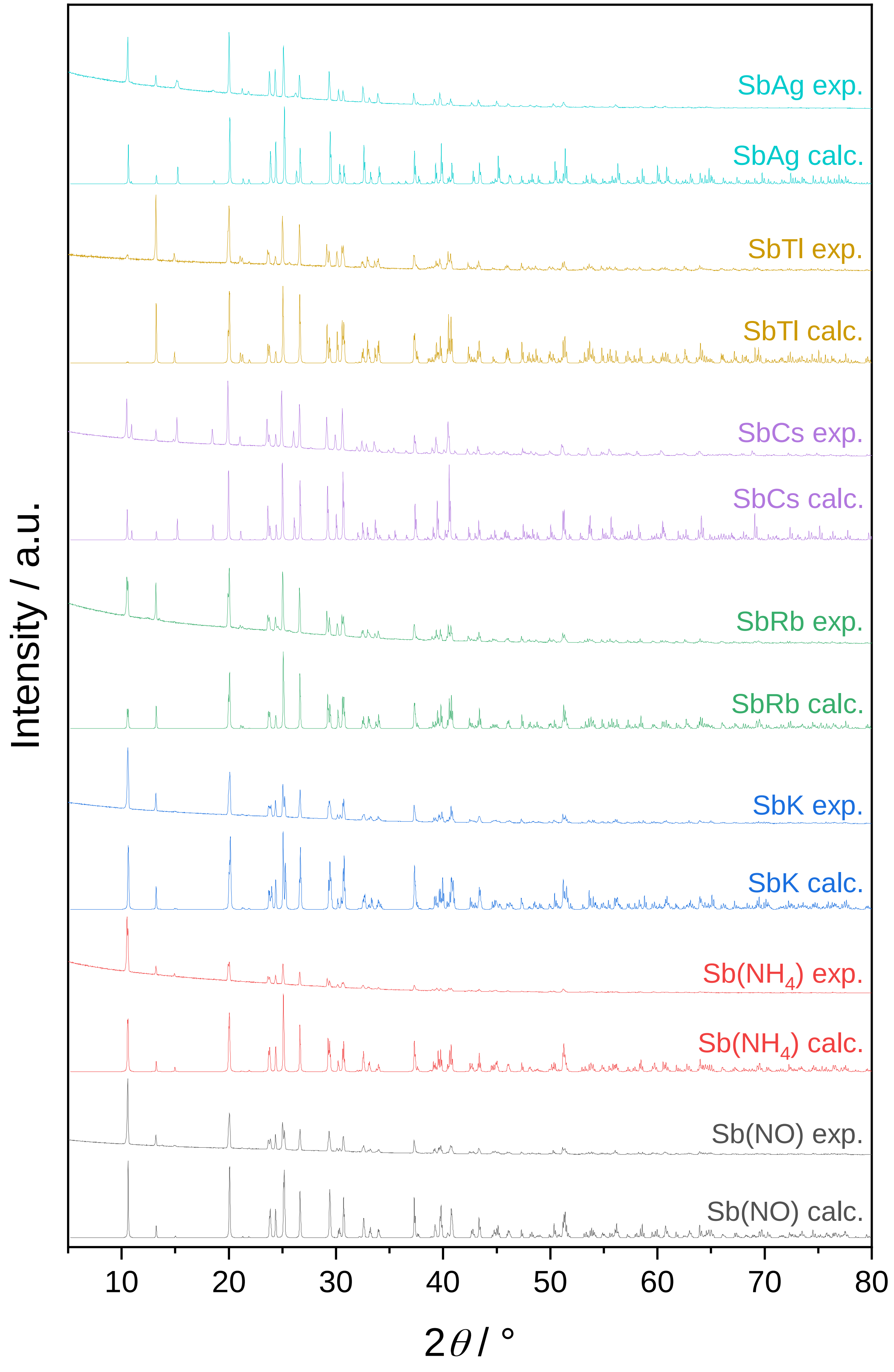

Figure S3. Calculated and experimental PXRD patterns of $SbX[B(SO_4)_2]_4$ (X = NO^+, NH_4^+, K^+, Rb^+, Cs^+, Tl^+, Ag^+).

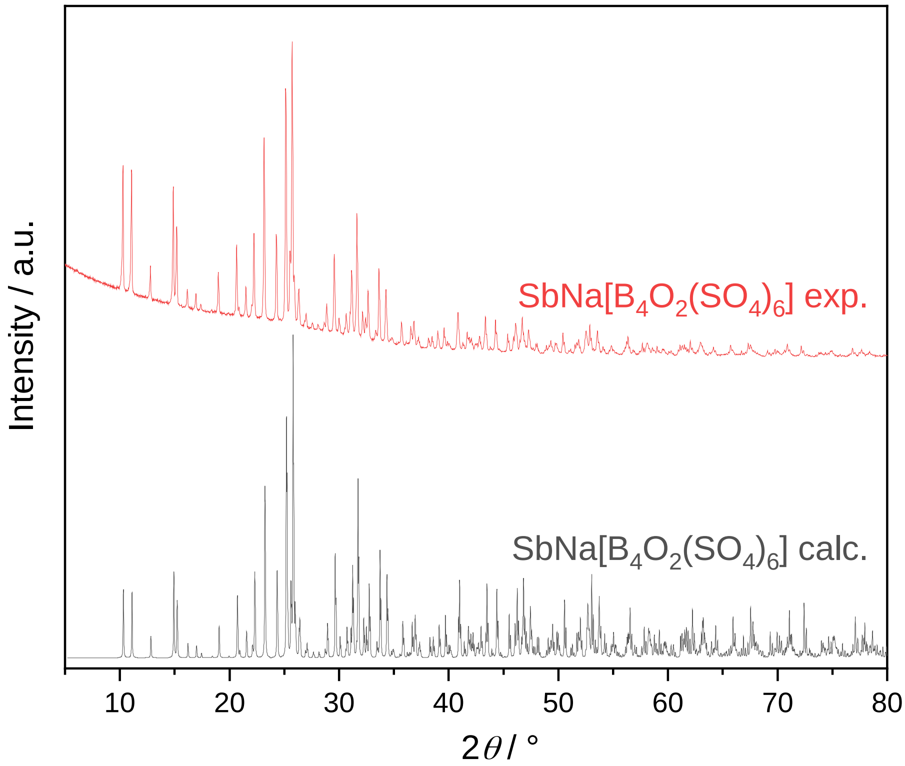

Figure S4. Experimental (red) and simulated (grey) XRD patterns of SbNa[$B_4O_2(SO_4)_2$]$_4$.

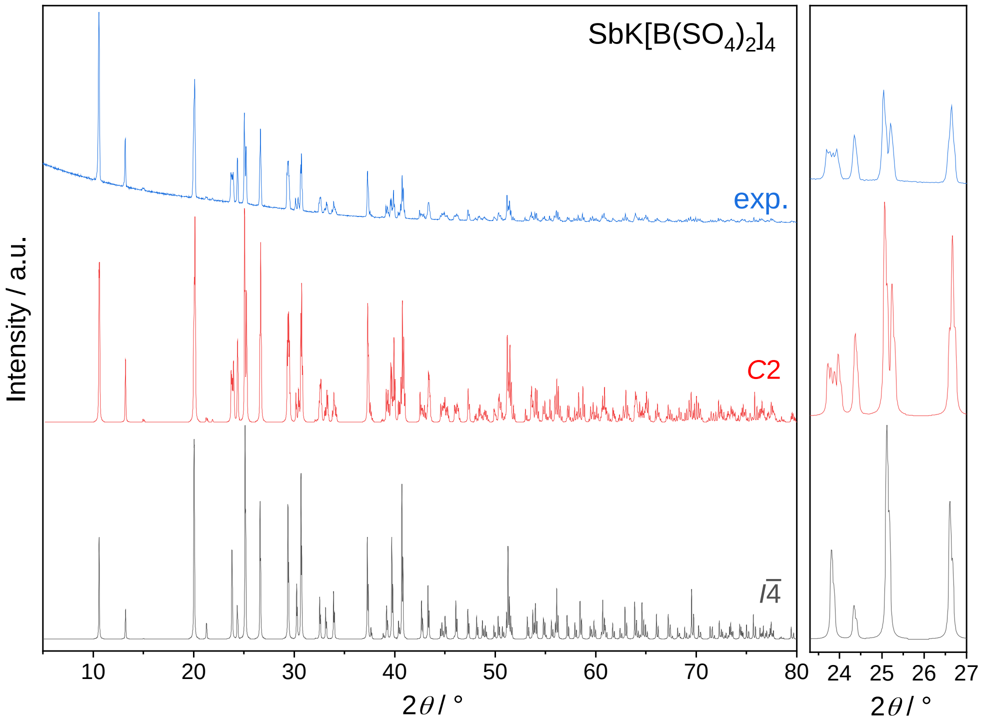

3

Figure S5. Experimental (blue) and two simulated XRD patterns (*C*2: red; *I*$\bar{4}$: grey) of SbK[B(SO_4)$_2$]$_4$ with a magnification between 23.5° and 27° 2*θ*.

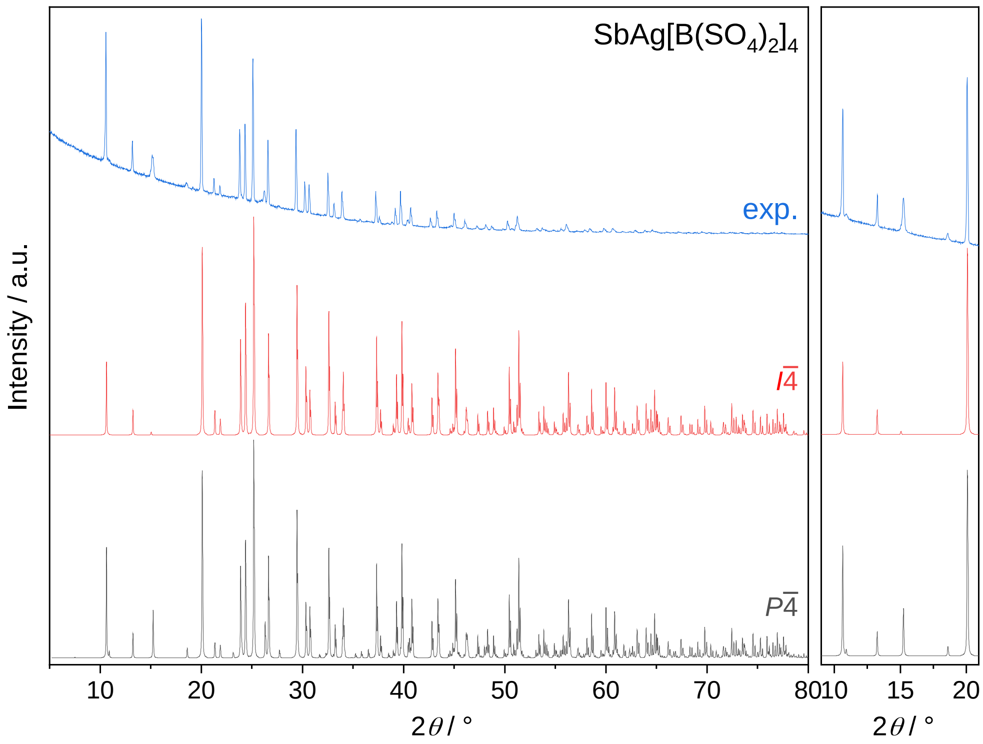

Figure S6: Experimental (blue) and two simulated XRD pattern ($I\bar{4}$: red; $P\bar{4}$: grey) of $SbAg[B(SO_4)_2]_4$ with a magnification between 8° and 21° 2θ.

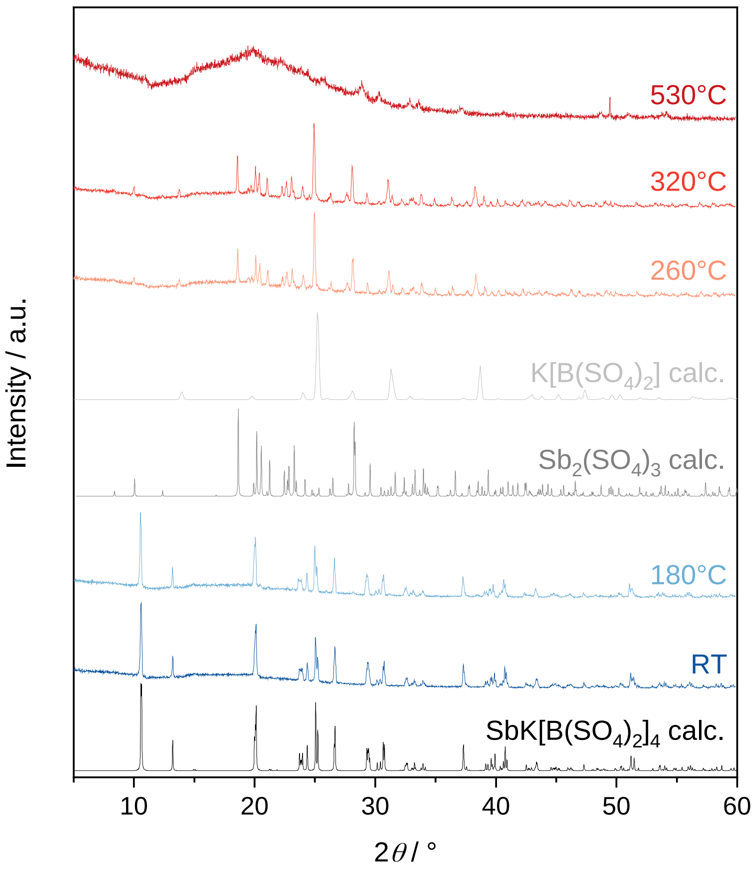

Figure S7. TPPXRD pattern of $SbK[B(SO_4)_2]_4$.d – d

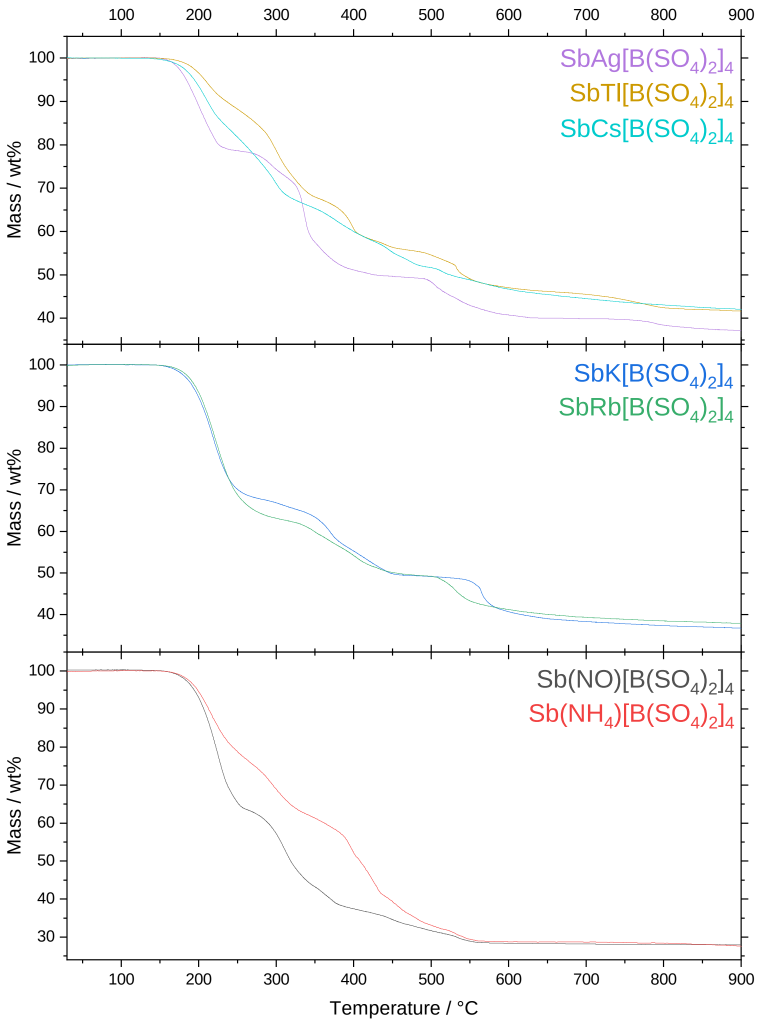

Figure S8: Thermogravimetric analyses of $SbX[B(SO_4)_2]_4$ (X = K^+, Rb^+, Cs^+, Ag^+, Tl^+, NO^+, NH_4^+) separated on three diagrams. For a better overview the graphs with similar shape were put into one diagram.

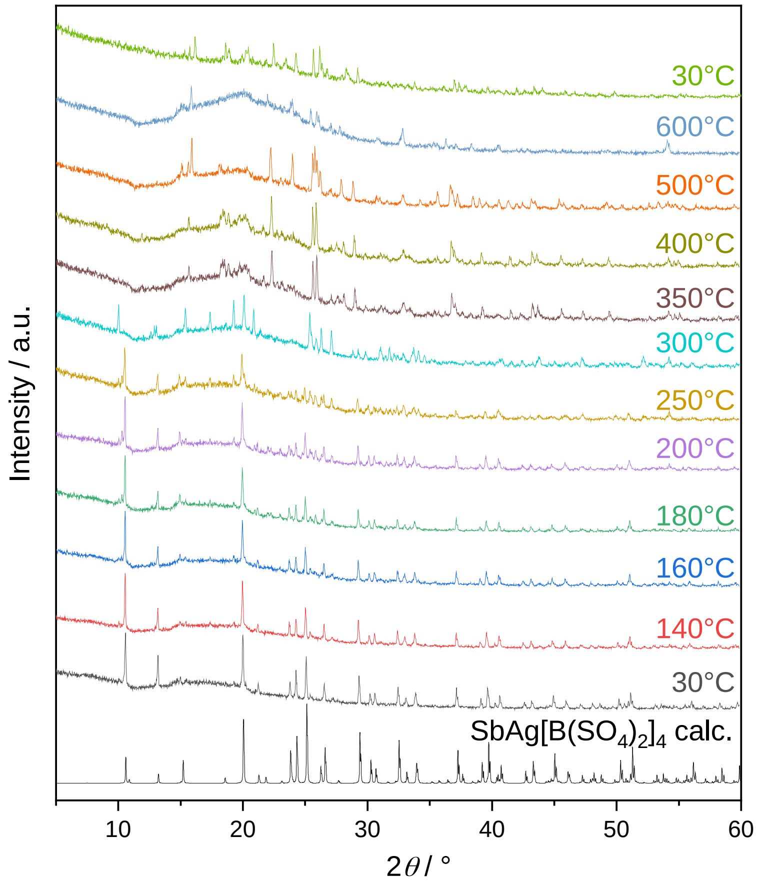

Figure S9. TPPXRD of $SbAg[B(SO_4)_2]_4$.

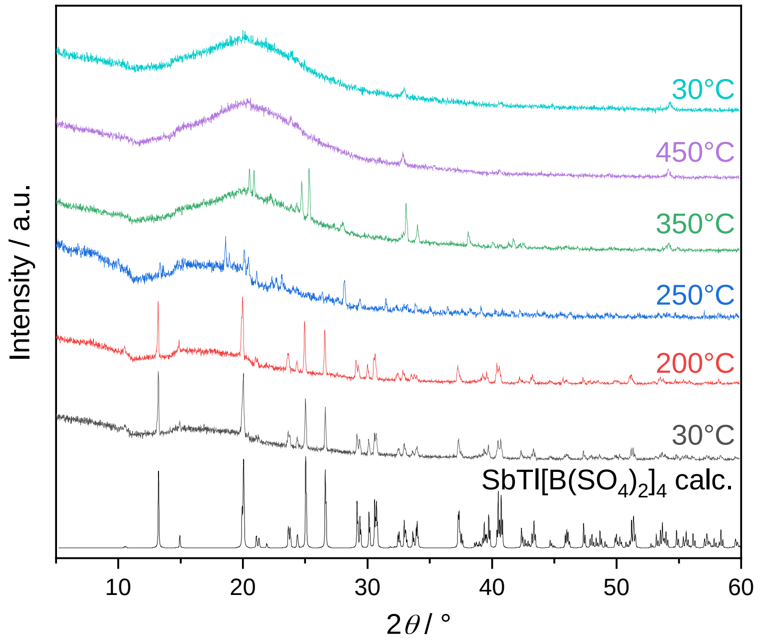

Figure S10. TPPXRD of $SbTl[B(SO_4)_2]_4$.

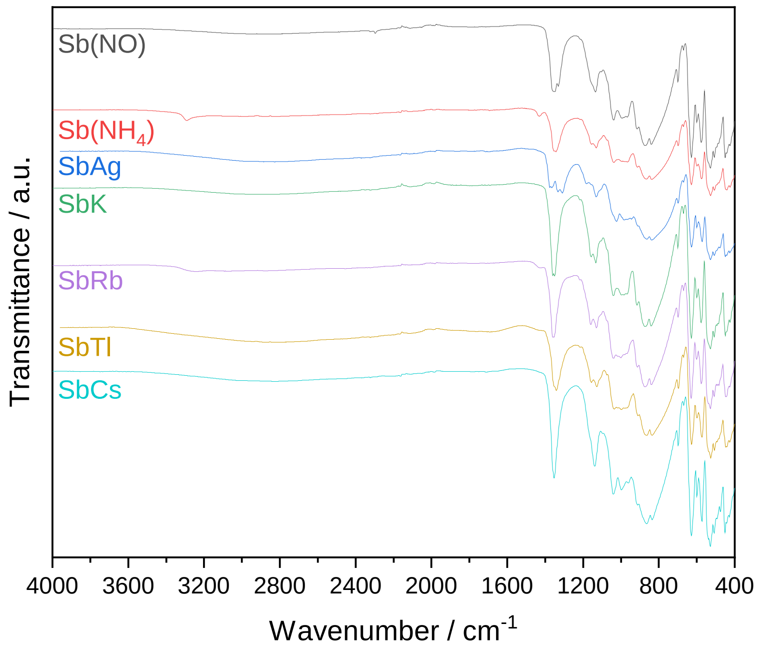

Figure S11. Full infrared spectra of $SbX[B(SO_4)_2]_4$ (X = K^+, Rb^+, Cs^+, Ag^+, Tl^+, NO^+, NH_4^+) in the range between 4000-400 cm^{-1}.

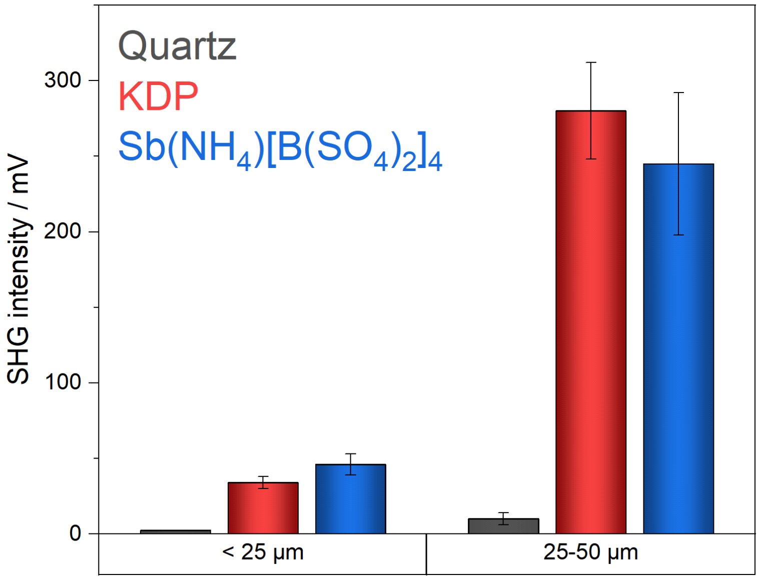

Figure S12. SHG intensities of $Sb(NH_4)[B(SO_4)_2]_4$, the reference material KDP and quartz recorded for the grain size range <25 µm and 25-50 µm.

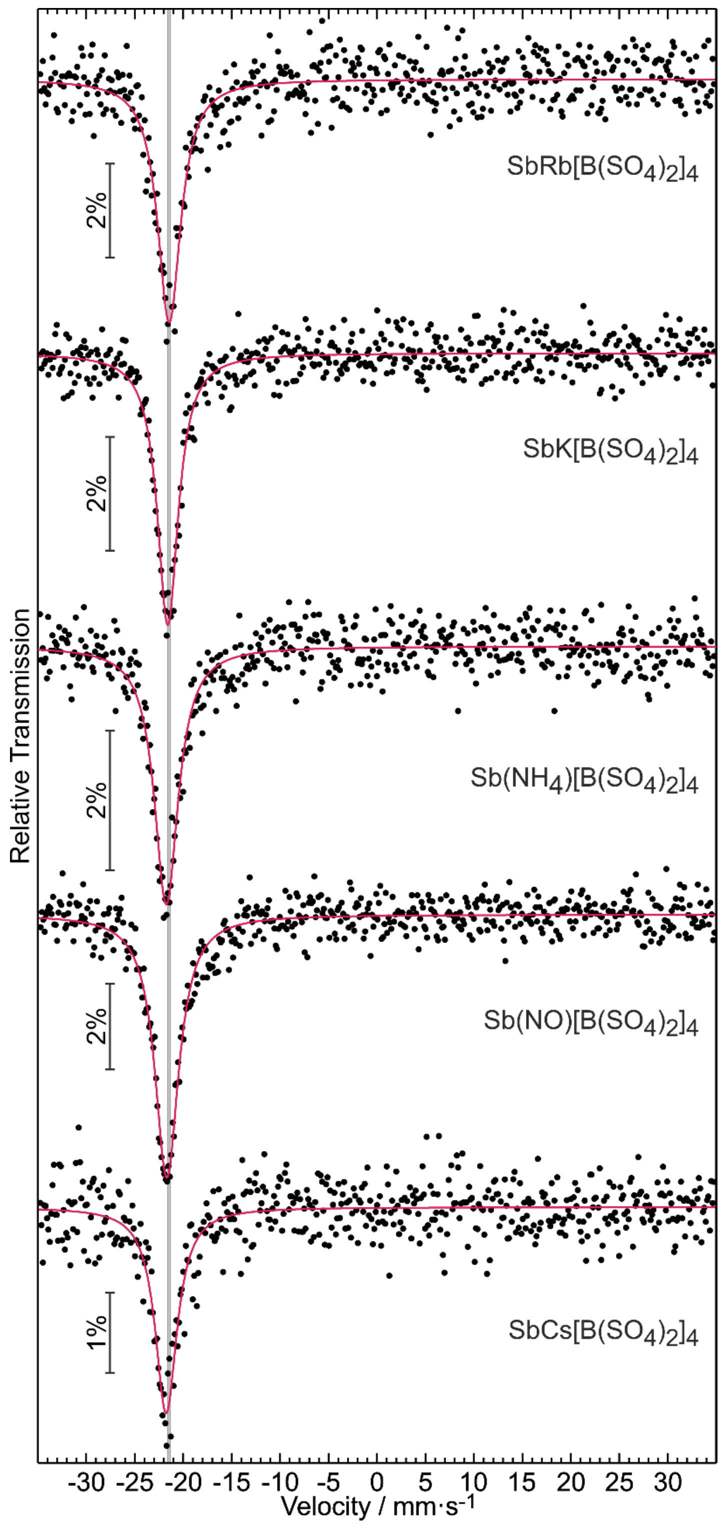

Figure S13. Experimental (data points) and simulated (red line) ^{121}Sb Mössbauer spectra of the $SbX[B(SO_4)_2]_4$ (X = Rb^+, K^+, NH_4^+, NO^+, Cs^+) samples at 78 K. The greyish line serves as a guide to the eye.

References

[1] Bruker, *SADABS*, Bruker AXS Inc., Madison, Wisconsin, USA, **2012**.
[2] G. M. Sheldrick, "A short history of SHELX", *Acta Crystallogr. A* **2008**, *64*, 112.
[3] G. M. Sheldrick, "Crystal structure refinement with SHELXL", *Acta Crystallogr. C* **2015**, *71*, 3.
[4] G. G. Long, J. G. Stevens, L. H. Bowen, "^{121}Sb Mössbauer Spectra of Antimony Oxides", *Inorg. Nucl. Chem. Lett.* **1969**, *5*, 799.
[5] T. Birchall, B. Della Valle, "The Non-existence of Sb_2S_5: a Mössbauer Spectroscopic Investigation of Some Antimony Chalcogenides and Oxides", *J. Chem. Soc. D* **1970**, 675.
[6] D. J. Stewart, O. Knop, C. Ayasse, F. W. D. Woodhams, "Pyrochlores. VII. The Oxides of Antimony: an X-Ray and Mössbauer Study", *Can. J. Chem.* **1972**, *50*, 690.
[7] L. Stievano, F. E. Wagner, H. W. Zanthoff, S. Calogero, "A ^{121}Sb Mössbauer Study of the Chemical State of Antimony in V–Sb–O Mixed-Oxide Catalysts for the Ammoxidation of Propane", *Hyperfine Int.* **2002**, *141*, 397.
[8] D. H. Moseley, R. Juneja, L. L. Daemen, I. Sergueev, R. Steinbrügge, O. Leupold, Y. Cheng, V. R. Cooper, L. Lindsay, M. K. Kidder, M. E. Manley, R. P. Hermann, "Vibrations and Phase Stability in Mixed Valence Antimony Oxide", *Inorg. Chem.* **2023**, *62*, 16464.
[9] R. A. Brand, *WinNormaos for Igor7 (version for Igor 7.010 or above: 01/03/2020)*, Universität Duisburg, Duisburg (Germany), **2020**.
[10] *CorelDRAW Graphics Suite 2017 (version 19.0.0.328)*, Corel Corporation, Ottawa, Ontario (Canada), **2017**.
[11] J. W. Zwanziger, "Computation of Mössbauer isomer shifts from first principles", *J. Phys.: Condens. Matter* **2009**, *21*, 195501.
[12] G. Kresse, J. Hafner, "*Ab initio* molecular dynamics for liquid metals", *Phys. Rev. B* **1993**, *47*, 558.
[13] G. Kresse, J. Furthmüller, "Efficiency of ab-initio total energy calculations for metals and semiconductors using a plane-wave basis set", *Comput. Mat. Sci.* **1996**, *6*, 15.
[14] G. Kresse, J. Furthmüller, "Efficient iterative schemes for ab initio total-energy calculations using a plane-wave basis set", *Phys. Rev. B* **1996**, *54*, 11169.
[15] G. Kresse, D. Joubert, "From ultrasoft pseudopotentials to the projector augmented-wave method", *Phys. Rev. B* **1999**, *59*, 1758.
[16] P. E. Blöchl, "Projector augmented-wave method", *Phys. Rev. B* **1994**, *50*, 17953.
[17] H. J. Monkhorst, J. D. Pack, "Special points for Brillouin-zone integrations", *Phys. Rev. B* **1976**, *13*, 5188.
[18] F. Tran, P. Blaha, "Accurate band gaps of semiconductors and insulators with a semilocal exchange-correlation potential", *Phys. Rev. Lett.* **2009**, *102*, 226401.
[19] T. W. D. Farley, W. Hayes, Hull, S. Hutchings, M. T., M. Vrtis, "Investigation of thermally induced Li^+ ion disorder in Li_2O using neutron diffraction", *J. Phys.: Condens. Matter* **1991**, *3*, 4761.
[20] E. Zintl, A. Harder, B. Dauth, "Gitterstruktur der Oxyde, Sulfide, Selenide und Telluride des Lithiums, Natriums und Kaliums", *Z. Elektrochem.* **1934**, *40*, 588.
[21] P. Touzain, F. Brisse, Caillet M., "Systèmes métaux alcalins–oxygène. 3ème Partie. Polymorphisme du monooxyde de potassium K_2O", *Can. J. Chem.* **1970**, *48*, 3358.
[22] A. Helms, W. Klemm, "Die Kristallstrukturen von Rubidium- und Cäsium-Oxyd", *Z. Anorg. Allg. Chem.* **1939**, *242*, 33.
[23] K.-R. Tsai, P. M. Harris, E. N. Lassettre, "The Crystal Structure of Cesium Monoxide", *J. Phys. Chem. B* **1956**, *60*, 338.
[24] H. Sabrowsky, "Zur Darstellung und Kristallstruktur von Tl_2O", *Z. Anorg. Allg. Chem.* **1971**, *381*, 266.
[25] P. Norby, R. Dinnebier, A. N. Fitch, "Decomposition of silver carbonate; the crystal structure of two high-temperature modifications of Ag_2CO_3", *Inorg. Chem.* **2002**, *41*, 3628.
[26] A. E. Whitten, B. Dittrich, M. A. Spackman, P. Turner, T. C. Brown, "Charge density analysis of two polymorphs of antimony(III) oxide", *Dalton Trans.* **2004**, 23.
[27] G. E. Gurr, P. W. Montgomery, C. D. Knutson, B. T. Gorres, "The crystal structure of trigonal diboron trioxide", *Acta Crystallogr. B* **1970**, *26*, 906.
[28] R. Pascard, C. Pascard-Billy, "Structure précise de l'anhydride sulfurique", *Acta Crystallogr.* **1965**, *18*, 830.
[29] J. Horakh, H. Borrmann, A. Simon, "Phase Relationships in the N_2O_3/N_2O_4 System and Crystal Structures of N_2O_3", *Chem. Eur. J.* **1995**, *1*, 389.
[30] R. Boese, N. Niederprüm, D. Bläser, A. Maulitz, M. Y. Antipin, P. R. Mallinson, "Single Crystal Structure and Electron Density Distribution of Ammonia at 160 K on the Basis of X-ray Diffraction Data", *J. Phys. Chem. B* **1997**, *101*, 5794.
[31] P. R. Mercier, J. Douglade, J. Bernard, "Structure Cristalline des $Sb_2O_3.3SO_3$", *Acta Crystallogr. B* **1976**, *32*, 2787.
[32] Devort J. P., Friedt J. M., "^{121}Sb Mössbauer Spectroscopy in alkali antimony(V) Hexafluorides", *Chem. Phy. Lett.* **1975**, *35*, 423.
[33] J. G. Ballard, T. Birchall, D. R. Slim, "Preparation of Antimony(V) Trichloride Difluoride and its Characterization by Means of X-ray Crystallography, Antimony-121 Mössbauer, and Raman Spectroscopy", *J. Chem. Soc., Dalton Trans.* **1977**, *15*, 1469.
[34] R. A. Pruitt, S. W. Marshall, C. M. O'Donnell, "Mössbauer Spectroscopy in Group-III Antimonides", *Phys. Rev. B* **1970**, *2*, 2383.
[35] S. L. Ruby, G. M. Kalvius, G. B. Beard, R. E. Snyder, "Interpretation of Mössbauer Measurements in Tin and Antimony", *Phys. Rev. B* **1967**, *159*, 239.
